# Development of Analytical Strategies for the Determination of Olive Fruit Bioactive Compounds Using UPLC-HRMS and HPLC-DAD. Chemical Characterization of *Kolovi* Lesvos Variety as a Case Study

**DOI:** 10.3390/molecules26237182

**Published:** 2021-11-26

**Authors:** Ioannis Martakos, Panagiota Katsianou, Georgios Koulis, Elvira Efstratiou, Eleni Nastou, Stylianos Nikas, Marilena Dasenaki, Michalis Pentogennis, Nikolaos Thomaidis

**Affiliations:** 1 Laboratory of Analytical Chemistry, Department of Chemistry, National and Kapodistrian University of Athens, Panepistimiopolis Zographou, 15771 Athens, Greece; johnmrtk@chem.uoa.gr (I.M.); katsianp@chem.uoa.gr (P.K.); georgekoulis@chem.uoa.gr (G.K.); elviraef@chem.uoa.gr (E.E.); elenanastou@chem.uoa.gr (E.N.); stelios_nikas@hotmail.com (S.N.); pentogmi@otenet.gr (M.P.); ntho@chem.uoa.gr (N.T.); 2 Laboratory of Food Chemistry, Department of Chemistry, National and Kapodistrian University of Athens, Panepistimiopolis Zographou, 15771 Athens, Greece

**Keywords:** olive fruit, antioxidants, phenolics, pigments, tocopherols, squalene, high performance liquid chromatography, mass spectrometry, *Kolovi* Lesvos variety

## Abstract

In this study, an overall survey regarding the determination of several bioactive compounds in olive fruit is presented. Two methodologies were developed, one UPLC-Q-TOF-MS method for the determination of olive fruit phenolic compounds and one HPLC-DAD methodology targeting the determination of pigments (chlorophylls and carotenoids), tocopherols (α-, β, -γ, δ-) and squalene. Target and suspect screening workflows were developed for the thorough fingerprinting of the phenolic fraction of olives. Both methods were validated, presenting excellent performance characteristics, and can be used as reliable tools for the monitoring of bioactive compounds in olive fruit samples. The developed methodologies were utilized to chemical characterize the fruits of the *Kolovi* olive variety, originating from the island of Lesvos, North Aegean Region, Greece. Twenty-five phenolic compounds were identified and quantified in *Kolovi* olives with verbascoside, hydroxytyrosol, oleacein and oleomissional found in significantly high concentrations. Moreover, 12 new bioactive compounds were identified in the samples using an in-house suspect database. The results of pigments analysis suggested that *Kolovi* variety should be characterized as low pigmentation, while the tocopherol and squalene content was relatively high compared to other olive varieties. The characterization of *Kolovi* olive bioactive content highlighted the high nutritional and possible economic value of the *Kolovi* olive fruit.

## 1. Introduction

Olive fruit and olive oil occupy a prominent place in the Mediterranean diet, not only for their unique taste and flavor but also for their high bioactive content. Olive is the fruit of the olive tree *(Olea Europea)*. The olive tree is mainly cultivated in the Mediterranean countries due to the temperate climate, which is ideal for cultivating and growing such trees [1]. Consequently, Mediterranean countries hold the first positions of farming, export, and consumption per year of table olives and olive oil. Specifically, according to the International Olive Council (IOC), Spain, Greece, Italy, Morocco, Egypt, Lebanon and Turkey, occupy the first seven places in cultivation, export and consumption for olive oil, as well as table olives (International Olive Council, IOC, https://www.internationaloliveoil.org/ accessed on 1 October 2021).

Olive products are a great source of bioactive compounds, divided into different chemical classes with various biochemical activities. Phenolic compounds (phenolic acids and derivatives, phenolic alcohols, flavones, lignans and secoiridoids) are natural antioxidants presented in olive tree products (olive fruit, olive oil and olive leaves) in different concentrations. These concentrations are influenced by cultivar, geographical origin, type of farming (biological or conventional), and tree treatment (watering, fertilizing, or both). All of the phenolic compounds have been reported as free-radical scavengers [2] and have been found to reduce the risk of atherosclerosis [3], as well as prevent the peroxidation of low-density lipoproteins (LDL), thus lowering the risk of cardiovascular diseases [1]. Furthermore, their radical scavenging activity is essential for increasing the products’ shelf-life [4]. The importance of these natural antioxidants is reflected in a health claim established by the European Union’s European Food Safety Authority (EFSA). This health claim reports that a concentration higher than 250 mg of hydroxytyrosol and its derivatives per kg of olive oil product, protects blood lipids from oxidative damage [5].

Notwithstanding, while the bioactive profile of olive oils of different varieties, geographical origins, and diverse agronomical backgrounds has been thoroughly studied over the past years, the same trend has not been observed regarding olive fruit and table olives. Filling this gap, it is of great significance to determine not only the total phenolic content of olives but also each phenolic compound individually as specific beneficial properties to human health have been attributed to many of them. For example, p-hydroxybenzoic, vanillic, caffeic, protocatechuic, syringic, and p-coumaric acids, oleuropein, and quercetin have shown antimicrobial activity by managing to contain the growth of certain fungi, bacteria and viruses [6], while oleuropein and hydroxytyrosol have been known to increase macrophage NO production [7].

Several studies have been reported in the previous years, presenting the identification and quantification of phenolic compounds in olive samples using reversed-phase high-performance liquid chromatography (RP-HPLC), coupled with either an Ultra-Violet (UV) or a Diode Array Detector (DAD) [7,8,9,10,11]. However, the application of High-Resolution Mass Spectrometry (HRMS) for the determination of olive phenolic content is still very scarce [12,13,14,15] even though the increased sensitivity, accuracy and mass resolution provided by HRMS technology enables the identification of new bioactive compounds, even trace-level metabolites, that would be impossible to detect using a low-resolution MS instrument or UV/DAD detectors [16,17]. Furthermore, the application of non-target screening strategies, such as suspect screening, could be a powerful tool for metabolic profiling and the thorough characterization of the olive fruit. As far as the extraction of phenolics is concerned, the solid-liquid extraction (SLE) method was used in the majority of the studies reported, using MeOH [9,18,19,20] or a mixture of methanol (MeOH): water [10,21] as the extracting agent.

Apart from phenolic compounds, olive fruit is also rich in other bioactive substances of high nutritional value, such as tocopherols, squalene, chlorophyllic and carotenoid pigments. Tocopherols are a group of lipid-soluble compounds that belong to the vitamin E complex. They are natural antioxidants presenting radical scavenging activity, resulting in the protection of body cells from oxidative damage, and they also have anti-aging and skin-protecting properties [22,23]. Squalene is a triterpene hydrocarbon that is present in all plants and it participates in the synthesis of sterols in plant and animal cells. Studies have shown anticancer and antioxidant activity [24,25]. Chlorophyllic and carotenoid pigments are compounds responsible for the color of the olive fruit. Chlorophyll and its derivatives (chlorophyll a and a’, pheophytin a and a’, pyropheophytin) are associated with the green color of the olive fruit. The color of the olive fruit is a significant characteristic that consumers often assess, and it is considered a quality marker [26,27]. Carotenoid pigments (β-carotene, lutein and other xanthophylls in minor percentages) have been reported as antioxidants with many other biochemical activities. *β*–carotene is a provitamin of vitamin A, while lutein has been linked with eye retinal protection and seems to possess anti-inflammatory properties [28,29]. Tocopherols, squalene, chlorophylls and carotenoids have been determined through the years with the use of RP-HPLC coupled with UV and DAD detectors [30,31,32,33,34,35], while Fluorescent detectors (FLD) were also used for tocopherols’ determination with both normal phase (NP-HPLC) and RP-HPLC [36,37]. In most of the methodologies reported, Olive fruit sample preparation consisted of an SLE step with *N, N*-dimethylformamide (DMF) for extraction of chlorophylls, and n-hexane for the extraction of tocopherols, carotenoids and squalene. Sagrattini et al. proposed solid-phase extraction (SPE) for sample cleaning and analyte extraction, with very good results [35]. Τo the best of the authors’ knowledge, so far, these compounds were determined individually and there is no method reported in the literature to achieve their simultaneous determination.

In this work, the development and validation of two novel analytical methodologies and workflows for determining bioactive compounds in fresh olive fruit are presented. An LC-QTOF-MS methodology, using both target and suspect screening workflows, was developed for the detection and identification of phenolic compounds and an HPLC-DAD method was developed to determine pigments, squalene and tocopherols, being the first time that a simultaneous determination of these compounds is carried out. These methods were used to characterize the chemical profile of the olive variety *Kolovi* of Lesvos. This variety is exclusively cultivated in Lesvos, the third biggest olive-growing region of Greece, and it is used to produce both olive oil and table olives. Previous studies of our research group [38,39,40,41] have revealed the excellent quality characteristics and health benefits of olive oils produced by olives of the *Kolovi* variety. As far as the authors’ knowledge, it is the first study performing a comprehensive *Kolovi* olive fruit characterization, indicating its exceptional bioactive profile and nutritional value.

## 2. Results and Discussion

### 2.1. Determination of Phenolic Compounds by LC-QTOF-MS

#### 2.1.1. Sample Preparation Optimization

##### Sample Pretreatment

The effect of freeze-drying on the phenolic content of the olive fruit was evaluated based on recovery experiments. The average recoveries of analytes, followed by the standard deviation (SD), are presented in Table S1 in the Supplementary Materials. Comparable results were obtained for most of the analytes in raw and freeze-dried samples, with recoveries ranging between 54–120% and 54–113%, respectively. However, it was noticed that freeze-drying improved the recoveries of the major olive fruit bioactive compounds (oleuropein, hydroxytyrosol, tyrosol, luteolin and rutin). Moreover, freeze-drying has been extensively used in the sample preparation of olives drupes intended for phenolic extraction [42,43,44] because it is characterized by several advantages, namely easy handling, longer storage until use, reduction in the cost of transportation and prevention of oxidative reaction of phenolic compounds. Thus, freeze-drying was performed as a preliminary step before the extraction procedure.

##### Selection of Extraction Solvent

The extraction ability of two different extraction media (100% MeOH and MeOH:H_2_O (80:20, *v*/*v*)) was tested in order to select the most appropriate for the Ultrasound-Assisted Extraction (UAE) of phenolic compounds from the olive matrix. The average recovery, followed by the SD for each spiked compound, is presented in Table S2 of the Supplementary Materials. According to these results, both extractants showed extraction efficiency higher than 70% for the majority of the analytes. Therefore, 100% MeOH was selected for two reasons: (a) it is an appropriate solvent for extraction via sonication, as no hydrogen peroxide neither large proportions of free radicals are formed when exposed to sonication, preventing the chemical degradation of phenols and (b) MeOH:H_2_O mixtures are usually responsible for the formation of emulsions [45].

##### Selection of Purification Step

The need for an extra purification step with the use of SPE was studied. Cartridges with three different sorbents were tested in that direction, namely C18, HLB (Hydrophilic-Lipophilic balance) and Isolute 101. Recovery experiments were performed and the average recovery for each analyte followed by the SD is presented in Table S3 in the Supplementary Materials. According to the results obtained, SPE using HLB cartridges showed the most satisfactory recovery yields for most analytes, especially for oleuropein, hydroxytyrosol, tyrosol, and rutin, which are the most common representative phenolic compounds in olive fruits, coming in agreement with previously reported studies [8,46]. Thus, SPE with HLB cartridges was selected as a purification step to reduce the matrix interferences of the olive matrix.

#### 2.1.2. Method Validation

Validation of the method ensued, as described in Section 3.3.2, and the results are presented in Table S4 of the Supplementary Materials. As shown, excellent linearity was achieved for the majority of the analytes with correlation coefficients greater than 0.99 (excluding diosmetin and tyrosol, which showed an R^2^ of 0.94 and 0.95, respectively). Regarding the sensitivity of the method, Limits of Detection (LODs) ranged from 0.15 mg/kg (4-hydroxybenzoic acid) to 3.6 mg/kg (tyrosol), while Limits of Quantification (LOQs) ranged from 0.45 mg/kg to 11 mg/kg, respectively. LODs and LOQs achieved with the proposed methodology were comparable to previously reported methods [12,44,45]. The precision of the method, repeatability and intermediate precision were estimated in terms of %Relative Standard Deviation (%RSD_r_ and %RSD_R,_ respectively). The RSD values were less than 20%, indicating the excellent precision of the proposed method. In addition, the developed method presented satisfactory results in terms of trueness as the recoveries for the majority of analytes ranged between 70 and 120% at all concentration levels. These results were similar to those of previous studies [8,12,44,45,47]. Finally, Matrix Effects (ME) of the developed methodology were calculated as the olive fruit is considered a challenging food matrix, primarily due to its complexity. Positive values of ME% indicate ion suppression, while negative values indicate ion enhancement. As presented in Table S4 of the Supplementary Materials, the matrix effects of analytes exceeded 20% only in low concentrations, indicating ion enhancement, while for higher concentrations, the matrix did not seem to interfere with the analyte determination (MEs fluctuating from −20% to +20%).

#### 2.1.3. Samples Results

##### Target Screening

The developed methodology was applied to analyze seven raw olive fruit samples from the *Kolovi* variety and determine their phenolic profile. A comprehensive database consisting of 42 phenolic compounds was composed for target screening (Table S5) and the identification was based on specific criteria described thoroughly below in Section 3.3.4. “Target Screening”. Consequently, 25 phenolic compounds were identified and quantified in *Kolovi* samples analyzed. As shown in Table 1, the main phenolic compounds in *Kolov*i olive fruits were verbascoside, oleacein, oleomissional and hydroxytyrosol. The concentration of these analytes ranging from 1255 to 14,223 mg/kg, 4 to 2447 mg/kg, 183 to 579 mg/kg and 187 to 928 mg/kg, respectively. Regarding verbascoside, it is the main hydroxycinnamic acid derivative of olive fruit and its concentration increases during ripeness. It is suggested that there is an inverse relationship between oleuropein and verbascoside. The partial enzymatic degradation of oleuropein during ripening can increase the concentration of verbascoside, as verbascoside is not detected in young raw olives [44]. The concentration of verbascoside in *Kolovi* variety was higher compared to olive cultivars originating from other Mediterranean countries, such as the *Tuscan* cultivar in Italy, the *Picual* and *Arbequina* cultivars in Spain and the *Sanulak* and *Gemlik* cultivars in Turkey, where verbascoside values <3000 mg/kg were referred [7,14,21,44]. The high concentration of verbascoside found in *Kolovi* samples emphasizes the beneficial properties of this fruit in human health, as many remarkable pharmacological activities, such as anti-inflammatory, antineoplastic, neuroprotective and wound healing were attributed to this analyte [48].

On the other hand, oleacein and oleomissional are oleuropein degradation products formed during the ripening period, contributing to the bitter taste of olive fruit. Since these compounds have recently been reported to exist in olive oil, there is only a published work about the quantitative determination of these two compounds in olive drupes and is referred to a Greek variety, namely *Koroneiki*. So, according to this research, the concentrations of oleacein and oleomissional were found at 1361.3 and 1599.7 mg/kg, respectively [42]. After comparing our results with the previously mentioned literature, it was revealed that oleomissional concentration was lower in all samples, while lower values of oleacein were detected in six out of seven *Kolovi* samples. Although their lower concentration levels compared to *Κoroneiki*, most of the samples contained a significant concentration of these two compounds contributing to the total bioactive content of the olive samples. Finally, hydroxytyrosol is the main biophenol in fresh olives and its concentration is increased during ripening and table olive processing. The analyte concentration in the studied samples was lower than other Portuguese or Turkish cultivars, such as *Sanulak* variety, while higher than Spanish cultivars, such as *Arbequina*. It is important to mention that the wide range in the concentrations of hydroxytyrosol and oleacein observed in our samples can be attributed to the samples’ different locations and harvesting times, as has also been reported in other studies [17,40,42]. The beneficial properties of these compounds have been proven, and therefore, they have been incorporated into the health claim of olive oil [5]. So, the high concentrations of hydroxytyrosol and its derivatives in the olive fruit of *Kolovi* variety confirm a product with a high nutritional value.

Other analytes detected in lower concentrations were oleuropein, luteolin and rutin. Oleuropein is the predominant phenolic compound in raw olives, mainly responsible for the bitter taste of fresh-picked olives. The concentration of this analyte is diminished during ripeness and falls to zero in fresh black olives or table olives. Significant variations were observed regarding the concentration levels of oleuropein in the analyzed samples (concentration range 3.5–855 mg/kg). This can be attributed to the different maturity stages of olive fruits studied. These results are comparable with those reported by other research groups that studied olive fruits of the *Manzanilla* variety or varieties originating from Turkey, such as *Gemlik*, with a mean concentration value of 300 mg/kg [8,44]. On the contrary, higher concentrations for oleuropein were presented in studies involving the analysis of Portuguese (*Macedo de Cavaleiros*, *Mirandela*, *Valpacos*, *Mogadouro and Figueira de Castelo Rodrigo, Fundao and Castelo Branco*), Turkish (Sarıulak) and Tunisian (*Chemlali*) varieties [21,46,49]. A great variance in the concentration levels was also noticed for luteolin, with concentrations varying from 48 to 450 mg/kg. This remark was also confirmed by other studies [17,21,44].

Another major compound in olive fruits was rutin, which was quantified in a 243 mg/kg mean concentration in the *Kolovi* variety. This concentration was lower than the respective one in Portuguese olive cultivars, while it was higher than other cultivars from Spanish or Turkish [19,46,50]. It is worth mentioning that rutin also demonstrates many pharmacological activities, such as cytoprotective, anti-carcinogenic, cardioprotective and vasoprotective, while it is connected to anti-Alzheimer and antiarthritic effects [51].

Finally, several analytes were detected in relatively low concentrations in the *Kolovi* olive samples, namely tyrosol, oleuropein aglycone and 10-hydroxy-decarboxymethyl-oleuropein aglycone. Τhe calculated concentrations of all these analytes were less than 100 mg/kg. Therefore, the existence of all these compounds increases the total bioactive content of the studied olive samples. So, consuming olives from the *Kolovi* variety results in many health benefits as it contains a high bioactive content.

##### Suspect Screening

One hundred thirty-one suspect compounds (Table S6) were screened in *Kolovi* olive samples using the suspect screening workflow and 12 new analytes were detected and tentatively identified, as shown in Table 2. The compounds determined belong to different classes of phenolic compounds, such as phenolic glucosides, phenolic oleosides as well as phenolic acids and their derivatives. Specifically, five glucosides of flavones (three glucosides of luteolin, apigenin-7 glucoside and isorhoifolin), four phenolic oleosides (caffeoyl secologanoside, comselogoside, dihydrooleuropein and oleoside), one hydroxyphenylacetic acid (3,4 dihydrophenylacetic acid) and two hydroxycinnamic acid derivatives (isoverbascoside and betahydroxyacteoside) were tentatively identified in the studied olive samples. The identification of suspect compounds was performed following the workflow presented comprehensively in Section 3.3.4. “Suspect Screening” 

According to Table 2, the mass accuracy and the isotopic fitting for all analytes were below 2 mDa and 50 mSigma, respectively. Furthermore, for most analytes, the difference between the experimental and the predicted retention time was below 1.8 min apart from beta-hydroxyacteoside and isoverbascoside, which was higher. Although these analytes belong to the model’s applicability domain, their predicted RT cannot be considered reliable. Moreover, the predicted retention time could not be calculated for some analytes, as mentioned in Section 3.3.4 “Suspect Screening”. The identification of the analytes was achieved either by comparing the experimental MS/MS fragments with the ones found in mass spectral libraries, such as Massbank, Mona and Metlin [52] or by comparing the experimental MS/MS fragments with the ones produced by an in-silico fragmentation tool, such as Metfrag [53].

**Figure 1:**
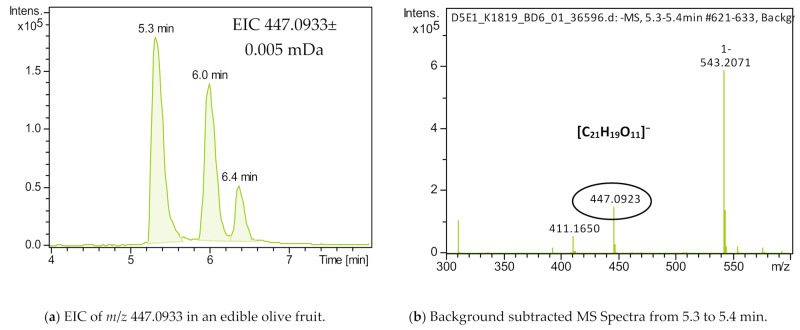

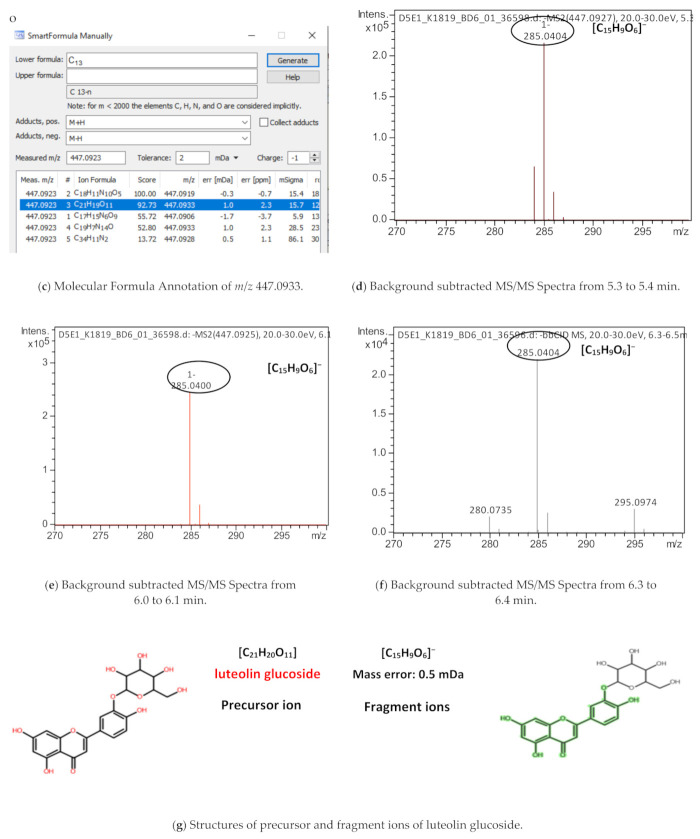

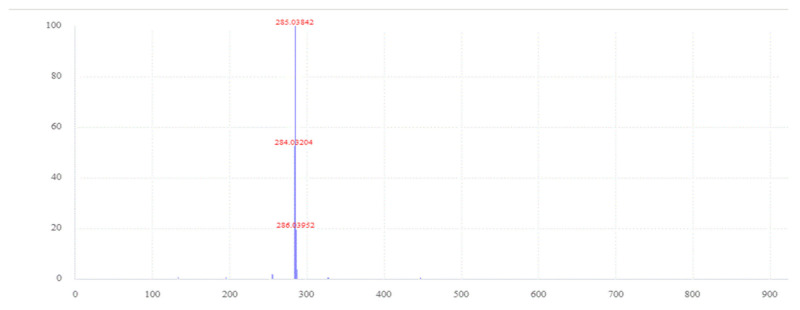
Identification procedure for luteolin glucoside isomers (**a**) EIC of *m*/*z* 447.0933 ( ± 0.005 mDa) in an olive sample; (**b**) Background subtracted MS spectrum from 5.3 to 5.4 min; (**c**) Molecular formula annotation of *m*/*z* 447.0933; (**d**) Background subtracted MS/MS spectrum from 5.3 to 5.4 min; (**e**) Background subtracted MS/MS spectrum from 6.0 to 6.1 min; (**f**) Background subtracted MS/MS spectrum from 6.3 to 6.5 min; (**g**) Structures of precursor and fragment ions of luteolin glucosides; (**h**) Riken Plant Specialized Metabolome Annotation (PlaSMA) Authentic Standard Library RIKENPlaSMA005600.

The identification workflow followed for the identification of luteolin glucoside isomers was thoroughly described below. Firstly, the extracted ion chromatogram of 447.0933 corresponding to the mass of the pseudomolecular ion of luteolin glucoside was created using the software Data Analysis 4.4 (Bruker Corporation, Billerica, MA, USA), as shown in Figure 1a, and three peaks were revealed. Next, the background-subtracted MS spectrum (Figure 1b) was meticulously examined to exclude that the *m*/*z* of interest is not formed as an in-source fragment by any other *m*/*z* existing in the mass spectrum. Based on the Smart Formula Manually tool (Bruker Corporation), the most probable molecular formula was C_18_H_12_N_10_O_5._ However, this formula was rejected as no compound has ever been referred to in literature. Therefore, the second formula (C_21_H_20_O_11_) was accepted with a mass error less than 2 mDa and an isotopic fit less than 20 mSigma, as shown in Figure 1c. According to the literature, this formula corresponds to nine possible candidates namely luteolin glucoside isomers (luteolin-3-O-glucoside, luteolin-5-O glucoside, luteolin-7-O-glucoside, luteolin-4-O-glucoside, luteolin-6-O-glucoside and luteolin-8-O-glucoside), quercitrin, kaempferol-3 glucoside and cyaniding-3-β-galactoside.

In Figure 1d–f, the MS/MS spectra for each peak after background subtraction were examined, and it was revealed that fragment 285.0404 was the most abundant ion for all three peaks. Finally, a comparison of the experimental MS/MS spectrum with that, that existed in the MS/MS Riken Plant specialized Metabolome Annotation Authentic Standard Library, recovered from MoNA (Mass Bank of North America), was ensued (Figure 1h). Thus, it was confirmed that the three peaks were glucosides of luteolin and the fragment 285.0404 corresponds to the release of the glucoside moiety, yielding the flavone luteolin. According to the literature three glucosides of luteolin were dominant in olive fruits, namely luteolin-7-O-glucoside, luteolin-3-O-glucoside and luteolin-4-O-glucoside [16,54,55]. As reported previously in the literature, in reversed-phase liquid chromatography, luteolin-7-O-glucoside elutes first, followed by luteolin-4-O-glucoside and luteolin-3-O-glucoside [54,55]. These glucosides of luteolin have been reported to encounter in many different cultivars, such as *Pisciottana* in Italy, *Istrska belica* in Slovenia and *Bical* or *Cobrançosa* in Portugal [4,13,46,55]. Raw olives are a promising source of flavonoids and especially luteolin glycoside isomers. This can also be confirmed by the high abundance of these glucosides found in all samples of our study. It should be emphasized that during the ripening stage of olives, a degradation of the glucosides is carried out, leading to an increase in the concentration of the luteolin moiety. Among flavonoids, luteolin and its glucosides display the highest antioxidant activity with many beneficial effects on human health. Furthermore, recent studies present the potential anti-COVID-19 properties of luteolin by binding with a high affinity to the same sites of the main protease of SARS-CoV-2 as the control molecule [56].

Apart from luteolin glucosides, other analytes were also identified. First, other flavonoid glucosides identified in the studied samples were isorhoifolin, a diglucoside of apigenin and the apigenin-7 glucoside. Regarding the first one, the extracted ion chromatogram of the *m*/*z* 577.1563 was created, showing one peak with a retention time of 5.7 min, as shown in Figure S1 of the Supplementary Materials. The most probable molecular formula proposed by Smart Formula Manually was C_27_H_30_O_14_. According to literature, three possible candidates (pelargonidin-3 rutinoside, kaempferitrin and isorhoifolin) reported in olive matrices can correspond to this formula. The first two candidates were rejected after comparing the MS/MS spectra with those in the Bruker Sumner MetaboBASE Plant Library recovered from Massbank of North America. So, the corresponding compound was finally identified as isorhoifolin. Traces of the analyte were also referred to exist in several black Spanish olive varieties, according to Romero et al., [10]. In the case of the glucoside of apigenin, the same workflow was applied in which its MS/MS spectrum was compared to the same aforementioned library of MONA (Figure S2). So, two common fragments (268.0370 and 269.0455) were revealed from this comparison. According to literature data, this glucoside of apigenin was also found at a considerable amount in several cultivars of Portugal [46]. Degradation of these glucosides can lead to the formation of apigenin, an analyte with an insignificant concentration in the olive samples.

Furthermore, four secoiridoids, namely oleoside, comseologoside, caffeoyl-secologanoside and dihydrooleuropein, were tentatively identified in the raw samples of *Kolovi* variety. Secoiridoids are the most important class of phenolic compounds in olive fruits and include several analytes, with the most abundant being the bitter compound oleuropein. They are a combination of elenolic acid and phenolic alcohols, such as hydroxytyrosol or tyrosol. Several biological properties have been reported regarding secoiridoids, many of which are attributed to antioxidant and free radical scavenging activity [4,7,21].

Regarding oleoside, the extracted ion chromatogram of the *m*/*z* 389.1085 showed two peaks as presented in Figure S3 of the Supplementary Materials. Τhe molecular formula proposed by SmartFormula ([C_16_H_22_O_11_]) corresponds to the oleoside and its isomer secologanoside. According to the literature data [16] and the comparison of MS/MS fragments with that produced by in-silico fragmentation, the existence of these two compounds was confirmed. Since these compounds are stereoisomers, the rt prediction model cannot distinguish them. So, the elution order of the analytes cannot be found. However, it is important to mention that in some previous research in which RP chromatographic system was used, it is suggested that oleoside is eluted first and then the secologanoside [16,57].

Also, two esters of secologanoside, namely caffeoyl 6-secologanoside and comselogoside, were tentatively identified in the raw olive samples with molecular formulas [C_25_H_28_O_14_] and [C_25_H_28_O_13_], respectively (Figures S4 and S5). The MS spectra and the fragmentation profiles were studied, and a comparison of MS/MS spectra with the respective one obtained by Metafrag has ensued. The MSMS fragments of these analytes were also compared with literature data. So, the four common fragments of caffeoyl-6 secologanoside (161.0242, 281.0659, 389.1977 and 507.1492) [45] and the three common fragments of comselogoside (145.0291, 265.0715 and 491.1550) [42] led to their identification. These two compounds have been reported in different olive varieties, such as in *Oblica* originating from Croatia [45], in *Picual* and *Arbequina* grown in Spain [14], or in *Istrska belica*, the most widely cultivated olive variety in Slovenia [16]. It is also known that the concentration of these compounds is high in raw olives and decreased during the formation of table olives or during the maturation period [57]. This is confirmed by the high abundance of these analytes in all the studied samples.

Apart from these, dihydrooleuropein was assigned to the deprotonated mass 543.2071 with a molecular formula [C_29_H_36_O_13_] according to Figure S6. Τhis formula corresponds to two different compounds, namely dihydrooleuropein and 8 acetoxy-4 methoxypinoresinol-4 glucoside. After studying the MS spectrum and evaluating its fragments with those acquired by MetFrag, dihydrooleuropein was tentatively identified, as the other structure cannot explain them. Moreover, according to several articles [13,57], this compound is considered a basic analyte in raw olives, which is confirmed by its high intensity in the studied samples.

Furthermore, two hydroxycinnamic acid derivatives, namely isoverbascoside (isoacteoside) and beta hydroxyacteoside were tentatively identified in olive fruits of the *Kolovi* variety. Several studies have reported that hydroxycinnamic acid derivatives are an important class of phenolic compounds and can act as powerful antioxidants to protect important molecules from oxidation. They can also be used as indicators of the maturation of olives since a drop of oleuropein is accompanied by a rise of these compounds [58]. Regarding the first analyte, the extracted ion chromatogram of the deprotonated mass 623.1974 showed two peaks (Figure S7). These two peaks were attributed to verbascoside and its isomer isoverbascoside with a molecular formula [C_29_H_36_O_15_]. The compound eluted at 4.9 min has been already identified from target screening as verbascoside, and so the compound with retention time 5.3 min was annotated as isoverbascoside. A thorough study of the MSMS spectra of the two isomers revealed that these analytes have the same fragmentation pattern with two characteristic ions. It is worth mentioning that these two isomers were detected in all the analyzed samples in great abundance, while they have also been reported in many different olive cultivars, such as *Manzanilla* and *Cucco* in Australia [59], *Sanulak* in Turkey [21], *Picual* in China [60].

The second analyte, beta-hydroxyacteoside, was assigned to the *m*/*z* 639.1929 with a molecular formula [C_29_H_36_O_16_], as shown in Figure S8. According to literature, this formula corresponds to two possible candidates (suspensaside and beta-hydroxyacteoside), which exist in olives. The tentative identification was based on the thorough study of the MS spectrum and comparing the MS/MS fragments with that produced by in-silico fragmentation. Thus, the extracted ion chromatogram of this *m*/*z* corresponded to the second analyte, namely hydroxyacteoside, because the other analyte cannot explain the fragments. High intensity was recorded in all of the studied samples, but there is limited information about the presence of this analyte in other varieties.

Finally, a phenolic acid tentatively identified in *Kolovi* olives was 3,4 dihydroxyphenylacetic acid assigned to the *m*/*z* 167.0346 with a molecular formula [C_8_H_8_O_4_] (Figure S9). In this case, the identification was based on the comparison of the experimental MSMS fragments with MSMS spectra found in the Fiehn Lab HILIC Library recovered from Massbank of North America. So, the common fragment 123.0439 led to the tentative identification of this analyte. In this study, it was detected in all *Kolovi* samples but at a moderate concentration level. Apart from the *Kolovi* variety, the presence of this analyte has also been reported in other Greek olive types, such as *Tsakistes*, *Amfissas* and *Kalamon* [1].

### 2.2. Determination of Pigments, Tocopherols, Squalene by HPLC-DAD

#### 2.2.1. Method Validation

The proposed methodology for the determination of pigments, tocopherols, squalene by HPLC-DAD was validated, showing excellent validation parameters and high recoveries for all the compounds of interest. Linearity was excellent, as the correlation coefficient for all the compounds was above 0.99 (Table 3). Trueness, repeatability and intermediate precision were assessed by calculating the %recoveries and the %RSDs of the spiked samples, and they are presented in Table 4. As is shown, for all the targeted compounds, the %recoveries were between 75–120% and all %RSDs were lower than 15%. LOD and LOQ were calculated using Equations (1) and (2), and they are expressed in both mg/L (referring to the olive extract) and in mg/kg referring to the initial raw olive sample.

LOD = 3.3 × SD/b(1)

LOQ = 10 × SD/b(2)

where SD refers to the standard deviation of the low concentration sample, and b = slope of the calibration curve of each analyte.

#### 2.2.2. Samples Results

##### Pigments, Tocopherols, Squalene

The developed methodology was applied to seven monocultivar samples of fresh olive fruit of the *Kolovi* variety, originating from Lesvos, North Aegean Region, Greece. The results obtained are presented in Table 5.

As presented in Table 5, chlorophyll a is the main pigment compound present in fresh fruit samples of the *Kolovi* variety. Compared with other olive fruit varieties, the chlorophyll A content of *Kolovi* was six times greater than the one of olives from the *Carolea* variety of Italy [61] and lower than in *Manzanilla* and *Hojiblanca* olives [34]. This can be easily explained, as, besides the varietal characteristics, most of the samples analyzed in our study were already mature (green-to-purple, purple and black color) when harvested, with the harvesting date playing a key role in the pigment composition (Table S7) [27]. The relatively low chlorophyll profile of *Kolovi* olives leads to low concentrations of chlorophyllic compounds in *Kolovi* olive oils, giving them characteristic yellow color [41].

The sum of carotenoids found in *Kolovi* olive fruits ranged between ND-5.7 mg/kg with an average of 4.5 mg/kg. The sum of carotenoids and lutein and *β*-carotene individually are similar to those reported by María Roca et al. [62] for olive fruits of the *Picual*, *Arbequina* and *Sikitita* varieties (for fruits with similar harvesting conditions). Moreover, the sum of pigments present in the *Kolovi* variety is similar to those of *Arbequina, Blanquietta* and *Cornicabra*, for the same ripening stage [63].

α- and (β + γ)-tocopherol concentrations appear to be higher in the fresh fruit of the *Kolovi* variety, in comparison with fruits of *Manzanilla* variety [33] and *Arauco* variety [64]. Regarding othreekeek olive varieties, *Kolovi* olives present a significantly higher concentration of α-tocopherol in the olive flesh (pericarp), while the (β + γ)-tocopherols are slightly lower compared with the fruit of the *Conservolea* and *Halkidiki* varieties [65]. In comparison with the most known Greek olive variety, *Koroneiki*, *Kolovi’s* tocopherol content was relatively low [66], as well as compared to the *Carolea* variety [61]. Finally, squalene’s concentration in the *Kolovi* variety appears to be three times higher than in the *Arauco* variety cultivated in Argentina [64]. So far, squalene content has been mostly studied in table olives (fermented and processed), and thus, data for squalene in raw olive fruit samples are very scarce.

## 3. Materials and Methods

### 3.1. Chemicals and Standards

#### 3.1.1. Chemicals and Standards for Phenolic Analysis

All phenolic compound standards were of purity higher than 95% except for verbascoside (86%); 2,5-dihydroxybenzoic acid (gentistic acid), 3,4-dihydroxybenzoic acid (protocatechuic acid), 4-hydroxybenzoic acid, catechin, cinnamic acid, gallic acid, ferulic acid, p-coumaric acid, quercetin, vanillic acid, pinoresinol, syringic acid, taxifolin, salicylic acid, rutin, eriodictyol, diosmetin, sinapic acid, quinic acid, eudesmic acid, citric acid, neochlorogenic acid, oleuropein, homovanillic acid, chlorogenic acid, kaempferol and verbascoside were obtained from Sigma-Aldrich (Steinheim, Germany). Luteolin, hydroxytyrosol and 2-cis,4-trans-Abscisic acid were purchased from Santa Cruz Biotechnology (Santa Cruz, CA, USA), while tyrosol, caffeic acid, vanillin and apigenin were purchased from Alfa Aesar (Karlsruhe, Germany). Finally, some standards were not purchased, but they were acquired from the Department of Pharmacy. Particularly six compounds, namely oleuropein aglycone, oleomissional, oleacein, ligstroside aglycone, oleocanthal and oleokoronal, were isolated from olive oil.

The reagents and solvents used for the analysis of phenolic compounds by LC-ESI-QTOF-MS were of high analytical purity. MeOH and acetonitrilcanACN) LC-MS grade were purchased from Merck (Darmstadt, Germany), while ammonium acetate (purity 99.0% or higher) was purchased from Sigma-Aldrich. Furthermore, ultrapure water (18.2 ΜΩ cm^−1^) was provided by a Milli-Q water purification system (Direct-Q UV, Millipore, Bedford, MA, USA). MeOH HPLC grade, purchased from Fischer Scientific (Geel, Belgium), was used for the extraction procedure, while hexane for analysis grade from Merck (Darmstadt, Germany) was used in the clean-up step to remove lipids. Additionally, several cartridges were used for the purification of samples. Specifically, Oasis HLB 6 cc Vac cartridges (200 mg) and Sep-Pak C18 6 cc Vac Cartridges (500 mg) were provided from Waters Corporation (Bedford, MA, USA), while Isolute 101 (200 mg/3 mL) cartridges were provided by Biotage (Sweden). Finally, regenerated cellulose syringe filters (RC filters, pore size 0.2 μm, diameter 15 mm) were acquired from Phenomenex (Torrance, CA, USA).

A stock solution of 1000 mg/L was prepared in MeOH and stored at −20 °C in dark glass bottles for each standard compound. An intermediate mix working solution of 20 mg/L of all analytes was prepared by diluting the standard stock solution in MeOH. The working solution was diluted in MeOH: H_2_O 50:50 to construct a calibration curve at five concentration levels (0.5, 1, 2, 5 and 10 mg/L). Moreover, a matrix-matched calibration curve was constructed by spiking the target analytes in a blank olive extract prior to injection.

#### 3.1.2. Chemicals and Standards for Pigment, Tocopherols and Squalene Analysis

HPLC grade MeOH, ethanol (EtOHcannd ACN were obtained from Fischer Scientific (Geel, Belgium). 2-propanol (isopropanol, IPA), acetone and *N*, *N*-Dimethylformamide (DMF) HPLC grade were purchased from Honeywell (Offenbach, Germany). Standard compounds used in this study (lutein, chlorophyll a, *β-*carotene, α-tocopherol, γ-tocopherol, δ-tocopherol and squalene) were acquired from Sigma Aldrich (Stenheim, Germany). Upon the arrival of the standards, stock solutions containing 1000 mg/L of each compound were prepared individually, in MeOH for lutein, in EtOH for β-carotene and tocopherols, in chloroform for squalene and in acetone for chlorophyll. Stock solutions were stored in a freezer at −20°C. During the study, intermediate standard solutions of 100–500 mg/L were prepared in IPA. Mixed working solutions were prepared in various concentrations to construct the calibration curves used to quantify each analyte. Before injection, samples were filtered through Chromafil Regenerated Cellulose (RC) syringe filters, 0.22 μm, purchased from Macherey-Nagel (Düren, Germany).

### 3.2. Olive Fruit Samples

Seven olive fruit samples of the *Kolovi* variety were obtained from two different olive groves in Lesvos Island, Greece. The olive fruits were hand-picked between 30 October and 13 November 2018. The olive samples were kept in plastic bags at 4 °C and were transferred to the laboratory in a mini-fridge after a period of one to three days after harvesting. Upon arrival at the laboratory, the olive fruits were de-pitted and freeze-dried to remove their humidity. Then, the freeze-dried olives were blended into a fine powder using an electric blender, to achieve homogenous samples. The powdered samples were stored in glass containers and kept at −20 °C, protected from light.

### 3.3. Phenolic Compounds Determination

#### 3.3.1. Sample Preparation

##### Method Optimization

Extracting phenolic compounds from the olive matrix is difficult because olive fruits represent a natural matrix containing several compounds, such as lipids, sugars, and polysaccharides [3,8]. Thus, an efficient analytical protocol is crucial for the accurate assessment of phenolic compounds in olives. Three critical parameters of the sample preparation methodology were examined and optimized: the sample pretreatment, the extraction media and the purification step of the proposed methodology. Different conditions were tested, and the recoveries of selected phenolic compounds (apigenin, caffeic acid, chlorogenic acid, cinnamic acid, eriodictyol, ferulic, hydroxytyrosol, kaempferol, luteolin, naringenin, oleuropein, p-coumaric acid, pinoresinol, quercetin, rutin, salicylic, syringic acid, taxifolin, tyrosol, vanillic acid and vanillin) were evaluated. All experiments were performed in triplicate using fortified olive fruit samples at a 5 mg/kg concentration level.

Sample Pretreatment: A drying procedure is a necessary step for the direct extraction of phenolic compounds from the olive fruit, preventing microbial spoilage and enzymatic degradation of the analytes. Freeze-drying is a methodology extensively used in olive fruit analysis [7,45,67], and so its effect on phenolic compound determination was assessed by performing recovery experiments both in raw olive and in the freeze-dried sample.

*Selection of extraction media:* Two different extractants were tested, a mixture of MeOH: H_2_O 80:20, *v*/*v* and 100% MeOH. They have been both used extensively for phenolic compound extraction [2,8,16,46].

Purification of olive extract: Apart from phenolic compounds, olive fruit contains lipids, sugars and salts. Thus, purification of the olive extract is vital to reduce the matrix effect and increase the accuracy and selectivity of the method. Consequently, a purification step with hexane was employed to remove lipids and liposoluble compounds. Moreover, the applicability of SPE in the removal of polar compounds, such as salts and sugars was tested. Finally, optimization experiments were performed following four different protocols, one without performing SPE and three using different SPE cartridges (Oasis HLB, C18 and Isolute 101).

##### Final Sample Preparation Protocol

In the final optimized sample preparation protocol, 0.5 g of well homogenized freeze-dried olive sample was weighted, and 5 mL of MeOH was added. The sample was vortex-mixed and the extraction was performed using Ultrasound Assisted Extraction (UAE) for 15 min. After the extraction, the sample was shaken for 15 min in a mechanical shaker. Then it was centrifuged for 5 min at 40,000 rpm, and the supernatant was decanted in a glass tube. This procedure was repeated twice, the extracts were combined, and 5 mL of water was added. A clean-up step with the addition of hexane ensued to remove lipids and the purified extract was evaporated in a rotary evaporator till dryness. Then, the dry residue was reconstituted with a solution of 2 mL MeOH: H_2_O 20:80. Afterward, 3 mL of acidified water was added to reduce the percentage of organic phase in the sample extract to 8%. It is well known that a high organic percentage can affect the retention of the analytes in the HLB cartridge. Finally, the extract was loaded to an HLB cartridge, pre-conditioned with 5 mL methanol and 5 mL acidified water. The target analytes were eluted using 10 mL of MeOH, and the methanolic extract was evaporated at 40 °C under a gentle nitrogen stream till dryness. Next, the reconstitution was performed with 1 mL of MeOH: H_2_O (50:50, *v*/*v*) and the extract was filtered through a 0.22 µm RC syringe filter to be ready for injection in the chromatographic system.

#### 3.3.2. LC-HRMS Methodology

The experiments were conducted using UHPLC (Dionex UltiMate 3000 RSLC, Thermo Fisher Scientific, Waltham, MA, USA) coupled with a QToF mass spectrometer (Maxis Impact, Bruker Daltonics) in negative electrospray ionization mode. The separation was performed on an Acclaim RSLC C18 column (2.1 × 100 mm, 2.2 μm) from Thermo Fischer Scientific, thermostated at 30 °C, equipped with an Acquity UPLC BEH C18 VanGuard Pre-Column from Waters. The mobile phase consisted of 90% water-10% methanol containing 5 mM ammonium acetate (Solvent A) and 100% methanol containing 5 mM ammonium acetate (Solvent B). The gradient elution program followed is presented in Table S8 of the Supplementary Materials. ESI source operating settings were the following: capillary voltage of 3500 V; end plate offset of 500 V, nebulizer pressure of 2 bar (N_2_), drying gas flow rate of 8 L min^−1^ (N_2_) and drying temperature of 200 °C. Full scan mass spectra were acquired for each sample in an *m*/*z* range 50−1000 with a spectra rate of 2 Hz. Bruker’s broadband collision-induced dissociation mode (bbCID) was used, where MS and MSMS spectra are obtained in the same run using two different collision energies (4 eV and 25 eV, respectively). bbCID provides high MS sensitivity, enabling the determination of even low-concentration compounds; however, MSMS spectra can be noisy, perplexing structure elucidation. For this reason, another MS analysis was additionally performed using Bruker AutoMS mode, which is a Data Dependent Acquisition mode. In this mode, the five most abundant ions per MS scan are selected and fragmented, providing much clearer and compound-specific MSMS spectra and enabling the identification of unknowns. A QTOF-MS external calibration was performed before analysis with clusters of 10 mM sodium formate solution in a mixture of water/isopropanol (50:50), and also internal calibration was performed by calibrant injection at the beginning of each run (1st segment, 0.1−0.25 min).

#### 3.3.3. Method Validation

The method’s performance was fully evaluated in terms of linearity, precision, trueness, LODs and LOQs and matrix effects under the Eurachem guidelines. The validation set consisted of 28 analytes, namely abscisic acid, 2,5 dihydroxybenzoic acid, 4 hydroxybenzoic acid, apigenin, caffeic acid, cinnamic acid, citric acid, diosmetin, eudesmic acid, ferulic acid, hydroxytyrosol, luteolin, naringenin, oleuropein, p- coumaric acid, pinoresinol, quercetin, salicylic acid, sinapic acid, tyrosol, vanillic acid, eriodictyol, chlorogenic acid, rutin, syringic acid, quinic acid, verbascoside and homovanillic acid. The validation experiments were conducted using fortified olive fruit samples constructed by spiking the target analytes to an olive matrix containing low concentrations of these compounds. Τhis was achieved because the sample used had undergone a debittering process, and thus the concentration of phenolic compounds was reduced.

Two calibration curves were constructed to assess linearity: an external calibration curve prepared by diluting standard stock solutions in pure solvent (MeOH: H_2_O, 50:50 *v*/*v*) and a matrix matched calibration curve prepared by fortifying the target analytes in olive extract (after the extraction procedure). Both curves were constructed using five different concentration levels (0.5, 1.0, 2.0, 5.0 and 10 mg/L) for all analytes and the linearity was determined by the least-squares method calculating the correlation coefficient (r).

Accuracy is one of the critical parameters to be assessed for method validation and involves common systematic errors (bias). It is estimated through trueness and precision. Regarding the calculation of trueness, recovery experiments were performed by spiking the target analytes in olive fruit at three concentration levels (1.0, 4.0 and 20.0 mg/kg) and analyzing spiked samples using the final extraction procedure. Recovery values were evaluated by comparing the response of the fortified samples (spiking before the extraction) to the response of the matrix-matched standards at the same final concentration (olive extracts fortified with the analytes after the extraction). Concerning precision, it was composed of repeatability (intra-day precision) and intermediate precision (inter-day precision). Repeatability was estimated by analyzing six replicates of the fortified samples at three different concentration levels (1.0, 4.0 and 20.0 mg/kg during the same laboratory day. For the assessment of intermediate precision, the same experiment was conducted during two consecutive laboratory days.

The LODs and LOQs for each analyte were calculated from the matrix matched calibration curve, using the standard deviation of the response (Sy) and the slope of the calibration curve. Specifically, they were determined with the following formulas: LOD = 3.3*(Sy/S) and LOQ = 10*(Sy/S). Finally, matrix effects were calculated for each analyte by comparing its response in the matrix-matched standard, subtracting this analyte’s response in the blank sample, with that in a standard prepared in a solvent at three concentration levels (0.5, 2 and 10 mg/L). The formula which was used for the determination of the matrix effect for each analyte is the following:

Matrix Effect% = [1 − (Peak area in the matrix-matched standard − Peak area in the blank sample/Peak area in the standard)] × 100.

#### 3.3.4. Screening Strategies

##### Target Screening

An accurate mass target screening database was compiled and used to identify and quantify specific phenolic compounds in olive samples. As depicted in Table S5, this database included 42 phenolic compounds from different chemical classes, such as phenolic acids, phenolic alcohols, phenolic aldehydes and flavonoids, for which reference standards were available in our lab. However, there were three compounds: acetoxypinoresinol, 10 hydroxyoleuropein aglycone and 10-hydroxy-decarboxymethyl oleuropein aglycone for which no reference standards were available. The information included in this database was the analytes’ name, the retention time, the molecular formula, and the exact masses of pseudomolecular and qualifier ions. The identification of the target analytes in the olive samples was based on the following criteria: (a) the mass error of the precursor ions and the qualifier ions should be less than 5 mDa, (b) the isotoping fitting should be less or equal than 50 mSigma, with Bruker mSigma being a measure of the goodness of fit between the measured and the theoretical isotopic pattern, (c) the retention time should be ±0.2 min compared to the retention time of the compounds in the standards (d) at least two qualifier ions should be detected € signal to noise ratio threshold was set at 3, (f) minimum area threshold was 800 and minimum intensity threshold 200. All analytes were quantified using standard solution calibration curves, apart from the above-mentioned analytes, which were semi-quantified, using the calibration curves of pinoresinol, oleuropein and hydroxytyrosol, respectively. The target screening was performed using Data Analysis 4.4 and TASQ 1.4 software (Bruker Daltonics, Bremen, Germany) along with other tools, such as Bruker Compass Isotope Pattern and SmartFormula Manually.

##### Suspect Screening

An in-house suspect database consisted of 131 bioactive compounds which have been previously reported in the literature [1,2,4,7,8,9,10,12,13,16,19,20,21,42,43,45,47,54,57,58,59,68,69,70,71,72] (to exist in olive fruit was composed and is presented in Table S6. Several classes of compounds, such as triterpenic acids, triterpenic alcohols, organic acids and some pigments were included in the suspect list. Compound names, molecular formulas, adduct and fragment information, and the predicted retention times calculated by an in-house retention time prediction model [73] were recorded. However, as seen in Table S6, there were some analytes for which the predicted retention time was not measured because their curated chemical structures (SMILEs) were not found in any literature data or libraries. The parameters applied for the suspect screening procedure were the following: mass accuracy threshold of 2 mDa, isotopic fit below or equal to 50 (mSigma), ion intensity of more than 800 and a peak area threshold of 2000. Additionally, a comparison of MS/MS fragments with that found in mass spectral libraries, such as MassBank [52], or from in silico fragmentation tools, such as Metfrag [53], was performed. Moreover, the evaluation of the predicted retention time played a vital role, especially in the case of isomers/tautomers, which often have identical MS/MS fragmentation patterns.

### 3.4. Pigment, Tocopherol and Squalene Determination

For the simultaneous determination of pigments, tocopherols and squalene, a modified version of the method reported by Minguez-Mosquera and Garrido-Fernandez for the extraction of chlorophylls and carotenoids was used [74]. Briefly, 0.5 g of freeze-dried sample was accurately weighed in a 15 mL polypropylene tube (Capp, Denmark). 2.5 mL of DMF were added using a volumetric cylinder. The sample was put in a shaker for 10 min and centrifuged at 4000 rpm for another 10 min. DMF phase, containing chlorophylls and xanthophylls, was transferred to a new polypropylene tube. 2.5 mL of n-hexane were added to the sample, and the same procedure was followed. The hexane phase, containing tocopherols, squalene and carotenoids, was retrieved and mixed with the DMF phase from the first step. The combined extracts were evaporated under a gentle stream of nitrogen. The sample was reconstituted in 2.5 mL of a 50:50 acetone: IPA mixture, filtered through a 0.22 μm RC filter and 20 μL was injected into the HPLC system. The HPLC system and the chromatographic conditions were presented in detail in a previous paper of our group [41]. Identification of targeted compounds was based on retention time and spectral data of standard solutions. For the quantification of each compound, calibration curves were constructed. β- and γ-tocopherol, have been quantified as a sum, using the γ-tocopherol standard as proposed by Gliszczyńska-Świgło et al. [75].

Our goal was to simultaneously extract chlorophylls and carotenoids, as well as tocopherols and squalene, which are lipid-soluble molecules and are contained in the fatty phase of olives and also to minimize the amount of solvents used, the mass of sample needed and the time required for the analytical process. HPLC chromatograms of olive samples spiked with a known concentration of the analytes can be found in Figure S10 of the Supplementary Materials.

Method Validation

The developed method was validated in terms of linearity, accuracy, precision, LODs and LOQs. For the study of linearity, calibration curves were constructed using standard solutions of at least six different concentrations. Precision was assessed through repeatability and intermediate precision experiments and was expressed as %Relative Standard Deviation (%RSD). For the repeatability assessment, three subsamples of an olive fruit sample were spiked at three different concentration levels (β-carotene: 2.95–14.75–29.5 mg/kg, lutein: 3.00–6.00–9.00 mg/kg, α-tocopherol: 50–100–250 mg/kg, γ-tocopherol: 50–100–250 mg/kg, δ-tocopherol: 50–100–250 mg/kg, squalene: 500–1000–1500 mg/kg and chlorophyll: 25.0–50.0–100.0 mg/kg) and the samples were analyzed with the proposed methodology in six replicates, under the same laboratory conditions (same laboratory day, analyst, instrumentation). The same procedure was followed during two more analytical days, for the assessment of intermediate precision. For the assessment of accuracy, the %Recovery of the spiked samples was calculated.

For the estimation of LODs and LOQs, ten subsamples of the reference sample were spiked with low quantity of analytes (β-carotene: 0.25 mg/kg, lutein: 0.25 mg/kg, α- tocopherol: 5.0 mg/kg, γ-tocopherol: 3.0 mg/kg, δ-tocopherol: 3.0 mg/kg, squalene 40.0 mg/kg and chlorophyll: 0.5 mg/kg) and the abovementioned analytical protocol was followed. Due to squalene’s high concentration in olives, it was not possible to find a blank or at least a low-concentration sample that could be used for LOD and LOQ estimation. Thus, according to Eurachem validation guidelines [76], we used procedural blanks spiked with a low concentration of squalene instead of olive fruit samples. LOD and LOQ were calculated using the mathematical equation provided by Eurachem [76].

## 4. Conclusions

In this study, two methods for determining phenolic compounds by UPLC-QTOF-MS and pigments, tocopherols and squalene simultaneously by HPLC-DAD in fresh olive fruit samples, were developed and validated. The methods proved to be accurate, precise and sensitive for all studied analytes. The *Kolovi* olive was selected for this research because no data was available in the literature, although it is one of the most important varieties in Greece and especially in the North Aegean region. To achieve a comprehensive characterization of the *Kolovi* variety, two analytical platforms were used in order to determine a wide range of compounds.

Concerning the analysis of phenolic compounds, *Kolovi* variety can be characterized by a high bioactive content due to the presence of many antioxidant compounds. Verbascoside, oleacein, oleomissional and hydroxytyrosol were found in high concentration levels, while oleuropein, tyrosol, rutin and luteolin were detected at a considerable concentration in *Kolovi* samples. Specifically, it is important to highlight the high concentration of verbascoside found in this olive fruit compared to others referred to in literature. These compounds are characterized by many health-promoting properties, such as anti-inflammatory, antimicrobial and anticarcinogenic, protecting against several diseases. Notably, luteolin has been currently reported as an inhibitory agent against SARS COVID-2019. Furthermore, 12 new phenolic compounds were tentatively identified through suspect screening, providing more information about *Kolovi* olive. Specifically, five glucosides of flavones (luteolin glucoside isomers, apigenin-7 glucoside and isorhoifolin), four phenolic oleosides (caffeoyl-secologanoside, comselogoside, oleoside and dihydrooleuropein), one hydroxyphenylacetic acid (3,4 dihydroxyphenylacetic acid) and two hydroxycinnamic acid derivatives (isoverbascoside and beta-hydroxyacteoside) were also found in the studied samples. Thus, the total number of detected bioactive constituents can demonstrate the importance of this variety and its capacity to produce products with health-beneficial properties.

Regarding non-phenolic compounds, *Kolovi* variety can be characterized as low pigmentation due to the relatively low concentrations of Chlorophyll a, *β*-carotene and lutein [27]. On the other hand, the tocopherol content appears to be above those presented in other varieties, ranging from 54–96 with a mean of 79 mg/kg for α-tocopherol and 2.34–4.30 with a mean of 3.37 mg/kg for (β + γ)-tocopherols. Furthermore, δ-tocopherol has not been detected in any of the samples analyzed in this study, suggesting that the *Kolovi* variety’s olives do not contain δ-tocopherol. Finally, the squalene content was relatively high ranging from 489–734 with a mean of 614 mg/kg of the olive fruit.

## Figures and Tables

**Table 1:** Target screening results (mg/kg).

Analytes	*Kolovi* Olive Fruit Samples						
	Sample 1	Sample 2	Sample 3	Sample 4	Sample 5	Sample 6	Sample 7
1-acetoxypinoresinol	12	17	31	10	12	3.9	5.7
10-hydroxy decarboxymethyl oleuropein aglycone	4.9	23	2.0	15	17	374	214
10-hydroxyoleuropein aglycone	1.8	2.4	0.99	1.6	0.50	0.53	2.1
apigenin	0.037	0.19	0.85	ND	0.93	ND	ND
caffeic acid	0.59	1.7	0.50	0.59	0.56	2.1	1.9
elenolic acid	17	45	22	15	23	30	24
eriodictyol	2.3	n.d	4.4	3.1	13	0.95	1.1
hydroxytyrosol	266	760	928	277	392	208	187
lingstroside aglycone	3.9	7.3	5.8	ND	5.3	8.2	ND
luteolin	48	291	450	149	284	150	75
oleokoronal	13	16	8.0	9.0	1.8	7.3	11
oleuropein	811	855	324	7.0	3.5	10	5.4
oleuropein aglycone	13	82	16	84	87	114	54
quercetin	ND	0.58	ND	0.46	ND	1.5	ND
tyrosol	48	78	123	19	29	17	19
vanillic acid	3.2	ND	4.3	1.0	1.5	0.38	0.46
vanillin	ND	ND	ND	ND	ND	3.8	5
oleacein	4.0	512	115	12	388	2447	434
oleocanthal	0.73	9.3	2.5	ND	6.2	24	7.7
oleocanthalic acid	0.36	4.2	0.59	ND	5.1	17	6.2
oleomissional	462	579	265	395	183	223	362
p-coumaric acid	ND	1.1	1.7	1.0	1.7	2.3	0.93
rutin	254	376	324	142	195	234	177
salycilic acid	ND	0.58	ND	0.15	ND	ND	ND
verbascoside	1255	9895	8821	6479	8970		6263

**Table 2:** Suspect screening results.

Compound	Molecular Formula	Ion	Mass Error (mDa)	t_R_ Predicted (min)	t_R_ Experimental (min)	Isotopic Fitting (mSigma)	*m*/*z* (Fragment ions)	# of Samples Detected
3,4 dihydroxyphenylacetic acid	C_8_H_8_O_4_	[M-H]^−^	0.4	0.39	3.1	12	123.0439	7
beta hydroxyacteoside	C_29_H_36_O_16_	[M-H]^−^	0.8	7.79	4.3	50	161.0243; 179.0349; 459.1505; 529.156; 621.1807	7
luteolin-7-o-glucoside	C_21_H_20_O_11_	[M-H]^−^	1.0	6.61	5.3	16	285.0400	7
luteolin-3-o-glucoside	C_21_H_20_O_11_	[M-H]^−^	1.0	6.58	6.4	16	285.0400	7
luteolin-4-o-glucoside	C_21_H_20_O_11_	[M-H]^−^	1.0	6.54	6	16	285.0400	7
caffeoyl-6 secologanoside	C_25_H_28_O_14_	[M-H]^−^	0.7	-	4.2	28	161.0242; 281.0659; 389.1077; 507.1492	7
comselogoside	C_25_H_28_O_13_	[M-H]^−^	0.2	-	4.6	24	145.0291; 265.0715; 491.1550	7
dihydrooleuropein	C_29_H_36_O_13_	[M-H]^−^	1.2	-	F5.4	24	313.1287; 357.1186; 377.1448; 389.1447; 525.1971	7
isoverbasoside	C_29_H_36_O_15_	[M-H]^−^	0.7	8.49	5.3	14	161.02423; 461.1659	7
oleoside	C_16_H_22_O_11_	[M-H]^−^	0.2	2.91	1.8	8.8	165.0558; 183.0666; 209.0460; 345.1186	3
apigenin 7-glucoside	C_21_H_20_O_10_	[M-H]^−^	0.6	6.39	5.9	31	269.0455; 268.0470	7
isorhoifolin	C_27_ H_30_O_14_	[M-H]^−^	0.9	6.81	5.7	32	269.0453; 270.0483	7

#: number.

**Table 3:** Concentration range, Calibration curves, correlation coefficients, LODs and LOQs of each analyte studied.

Analyte	Concentration Range (mg/L)	Calibration Curve	Correlation Factor Can	LOD (mg/kg) (n = 10)	LOQ (mg/kg) (n = 10)
lutein	0.05–6.00	y = 207.24–x − 8.0363	0.998	0.15	0.51
β-carotene	0.30–4.00	y = 105.7–x − 6.7561	0.995	0.07	0.23
α-tocopherol	2.00–75.0	y = 7.496–x − 13.526	0.991	1.1	3.7
γ-tocopherol	0.60–10.0	y = 9.8181x + 3.0003	0.991	0.55	1.8
δ-tocopherol	1.00–24.0	y = 7.623–x − 0.2302	0.994	1.0	3.4
chlorophyll	0.50–15.0	y = 74.15–x − 6.949	0.996	0.21	0.71
squalene	150–700	y = 38.318x + 3137.2	0.9996	4.2	13.9

**Table 4:** %Recoveries and RSD for the analytes of interest.

	%RecoveryEvaluation of Accuracy			RSDrEvaluation of Repeatability			RSD_R_Evaluation of Intermediate Precision		
	Low Concentration (%R) (n = 18)	Medium Concentration (%R) (n = 18)	High Concentration (%R) (n = 6)	Low Concentration(%RSD) (n = 6)	Medium Concentration (%RSD) (n = 6)	High Concentration(%RSD) (n = 6)	Low Concentration (%RSD) (n = 18)	Medium Concentration(%RSD) (n = 18)	High Concentration(%RSD) (n = 18)
lutein	89.2	88.4	81.7	4.7	5.1	3.6	9.8	7.3	6.2
β-carotene	100.4	91.9	93.4	2.2	3.9	3.7	6.4	13.5	14.7
α-tocopherol	99.6	99.3	99.4	3.5	5.3	5.4	4.5	4.3	5.4
γ-tocopherol	97.6	93.0	89.3	3.2	2.5	9.5	12.0	6.7	6.8
δ-tocopherol	101.1	95.4	93.5	3.2	1.8	4.8	12.3	5.6	9.1
chlorophyll	88.3	88.0	83.7	5.9	5.1	4.8	9.0	5.8	5.5
squalene	93.2	84.3	81.0	6.7	7.0	4.2	11.2	9.5	10.8

**Table 5:** Results for pigments, tocopherols and squalene.

	Chlorophyll	Lutein	β-Carotene	α-Tocopherol	(β + γ)-Tocopherols	Squalene
	mg/kg Raw Sample					
Sample 1	12.6	2.39	2.08	54	2.34	634
Sample 2	11.8	2.71	1.88	96	3.29	489
Sample 3	12.2	2.95	1.92	84	3.41	659
Sample 4	12.0	3.29	2.39	95	2.89	632
Sample 5	8.2	2.33	1.39	57	3.60	734
Sample 6	9.3	<LOQ	<LOD	91	4.30	529
Sample 7	<LOQ	2.17	1.53	76	3.74	619
Average concentration	9.6	2.64	1.87	79	3.37	614
Concentration range	<LOQ −12.6	<LOQ −3.29	<LOD−2.39	54–96	2.34–4.30	489–734
SD*	3.7	0.66	0.72	16	0.58	76

For the estimation of average concentration and standard deviation, only results above the LOD and LOQ were used, SD*: Standard Deviation.

## Data Availability

Not available.

## Supplementary Materials

The following are available online, Figure S1. Identification data for the mass feature m/z 577.1602_5.7 min (Isorhoifolin), Figure S2. Identification data for the mass feature m/z 431.0984_5.9 min (apigenin-7 glucoside), Figure S3. Identification data for the mass feature m/z 389.1089_1.8 min (oleoside), Figure S4. Identification data for the mass feature m/z 551.1406_4.2 min (caffeoyl 6-secologanoside), Figure S5. Identification data for the mass feature m/z 535.1455_4.6 min (Comselogoside), Figure S6. Identification data for the mass feature m/z 543.2083_5.4 min (dihydrooleuropein), Figure S7. Identification data for the mass feature m/z 623.1981_5.3 min (Isoverbascoside), Figure S8. Identification data for the mass feature m/z 639.1929_4.3 min (beta-hydroxyacteoside), Figure S9. Identification data for the mass feature m/z 167.0350_3.1 min (3,4 dihydroxyphenylacetic acid), Figure S10. Chromatograms from the analysis of olive drupes. spiked with known amount of the analytes of interest. A: 410 nm (spiked with 25 mg/kg chlorophyll a). B: 450 nm (spiked with 3 mg/kg lutein and 2.95 mg/kg β-carotene). C: 295 nm (spiked with 50 mg/kg α-, γ-, and δ-tocopherols). D: 210 nm (spiked with 500 mg/kg squalene), Table S1. Recovery rate % and SD for each spiked compound in 2 different experiments, Table S2. Recovery rate % and SD for each spiked compound, Table S3. %Recovery rate (%R) and SD for each spiked compound, Table S4. Evaluation of linearity, repeatability, intermediate precision, trueness and selectivity for the determination of phenolic compounds in olive fruits, Table S5. Target list of phenolic compounds, Table S6. Suspect list of bioactive compounds encountered in olive samples, Table S7. Maturation stage of Kolovi samples, Table S8. LC gradient elution and flow rate program (UPLC-QTOF-MS).

## Sample Availability

Not available.
