# Supplementary material for: Development of Analytical Strategies for the Determination of Olive Fruit Bioactive Compounds Using UPLC-HRMS and HPLC-DAD. Chemical Characterization of Kolovi Lesvos Variety as a Case Study
Source: Molecules. 2021 Nov 26;26(23):7182. doi: 10.3390/molecules26237182 (PMC8659053; doi:10.3390/molecules26237182)
Supplement: Supplementary file 1 [file molecules-26-07182-s001.zip › molecules-1460133-SI.pdf]

Development of analytical strategies for the determination of olive fruit bioactive compounds using UPLC-HRMS and HPLC-DAD. Chemical characterization of Kolovi Lesvos variety as a case study.

Ioannis C. Martakos†, Panagiota Katsianou†, Georgios Koulis, Elvira Efstratiou, Eleni Nastou, Stylianos Nikas, Marilena Dasenaki, Michalis Pentogennis and Nikolaos Thomaidis

† These authors contributed equally to this work.

Electronic Supplementary Material

Table of contents

Table S1. Recovery rate % and SD for each spiked compound in 2 different experiments………………………… 4

Table S2. Recovery rate % and SD for each spiked compound……………………………………………………5

Table S3. %Recovery rate (%R) and SD for each spiked compound…………………………………………………6

Table S4. Evaluation of linearity, repeatability, intermediate precision, trueness and selectivity for the determination of phenolic compounds in olive fruits……………………………………………………………………………………7

Table S5. Target list of phenolic compounds………………………………………………………………………8

Table S6. Suspect list of bioactive compounds encountered in olive samples…………………………………………9

Figure S1. Identification data for the mass feature m/z 577.1602_5.7 min (Isorhoifolin)……………………………13

Figure S2. Identification data for the mass feature m/z 431.0984_5.9 min (apigenin-7 glucoside)………………….15

Figure S3. Identification data for the mass feature m/z 389.1089_1.8 min (oleoside)…………………………………16

Figure S4. Identification data for the mass feature m/z 551.1406_4.2 min (caffeoyl 6-secologanoside)……………..18

Figure S5. Identification data for the mass feature m/z 535.1455_4.6 min (Comselogoside)………………………….19

Figure S6. Identification data for the mass feature m/z 543.2083_5.4 min (dihydrooleuropein)……………………..20

Figure S7. Identification data for the mass feature m/z 623.1981_5.3 min (Isoverbascoside)…………………………21

Figure S8. Identification data for the mass feature m/z 639.1929_4.3 min (beta-hydroxyacteoside)…………………23

Figure S9. Identification data for the mass feature m/z 167.0350_3.1 min (3,4 dihydroxyphenylacetic acid)……….25

Table S7. Maturation stage of Kolovi samples……………………………………………………………………..26

Table S8. LC gradient elution and flow rate program (UPLC-QTOF-MS)……………………………………………26

Figure S10. Chromatograms from the analysis of olive drupes. spiked with known amount of the analytes of interest. A: 410 nm (spiked with 25 mg/kg chlorophyll a). B: 450 nm (spiked with 3 mg/kg lutein and 2.95 mg/kg β-carotene). C: 295 nm (spiked with 50 mg/kg α-, γ-, and δ-tocopherols). D: 210 nm (spiked with 500 mg/kg squalene)…………………………………………………..….27

Table S1. Recovery rate % and SD for each spiked compound in 2 different experiments

	**Raw olive fruit**	**Freeze-dried olive fruit**
**Compound**	**R% ± SD (n=3)**	**R% ± SD (n=3)**
Apigenin	64 ± 12	71 ± 9.3
Caffeic acid	72 ± 10	92 ± 5.3
Chlorogenic acid	114 +17	81 ± 17
Cinnamic acid	114 ± 14	94 ± 13
Eriodictyol	57 ± 9.5	81 ± 6.6
Ferulic acid	106 ± 8.2	85 ± 8.9
Hydroxytyrosol	93 ± 14	102 ± 15
Kaempferol	75 ± 16	54 ± 14
Luteolin	77 ± 19	96 ± 17
Naringenin	89 ± 14	60 ± 10
Oleuropein	86 ± 12	101 ± 8.8
P-coumaric acid	64 ± 11	96 ± 6.4
Pinoresinol	114 ± 20	94 ± 19
Quercetin	54 ± 11	63 ± 6.9
Rutin	72 ± 20	98 ± 19
Salicylic acid	120 ± 8.3	92 ± 7.1
Syringic acid	78 ± 9.3	86 ± 8.8
Taxifolin	80 ± 11	59 ± 11
Tyrosol	117 ± 12	113 ± 9.6
Vanillic acid	101 ± 8.7	105 ± 8.2
Vanillin	101 ± 13	72 ± 12

Table S2. Recovery rate % and SD for each spiked compound

	MeOH: H2O (80:20)	100% MeOH
**Analyte**	**R% ± SD (n=3)**	**R% ± SD (n=3)**
Apigenin	71 ± 9.3	62 ± 9.9
Caffeic acid	92 ± 5.6	98 ± 3.9
Chlorogenic acid	81 ± 17	83 ± 16
Cinnamic acid	94 ± 13	91 ± 11
Eriodictyol	81 ± 6.6	70 ± 6.9
Ferulic acid	85 ± 8.0	88 ± 8.2
Hydroxytyrosol	102 ± 12	102 ± 11
Kaempferol	44 ± 15	45 ± 16
Luteolin	96 ± 17	94 ± 17
Narinegin	40 ± 10	46 ± 9.4
Oleuropein	101 ± 8.8	111 ± 5.8
P-coumaric acid	96 ± 6.4	101 ± 3.1
Pinoresinol	94 ± 19	117 ± 13
Quercetin	53 ± 6.9	44 ± 8.6
Rutin	98 ± 19	101 ± 12
Salicylic acid	92 ± 7.5	94 ± 4.9
Syringic acid	86 ± 8.8	88 ± 7.3
Taxifolin	39 ± 11	39 ± 5.7
Tyrosol	113 ± 9.6	116 ± 5.9
Vanillic acid	105 ± 8.2	125 ± 8.8
Vanillin	72 ± 12	94 ± 13

Table S3. %Recovery rate (%R) and SD for each spiked compound

	Without SPE	SPE (HLB)	SPE (C18)	SPE (ISOLUTE)
**Analyte**	**R% ± SD (n=3)**	**R% ± SD (n=3)**	**R% ± SD (n=3)**	**R% ± SD (n=3)**
Apigenin	42 ± 9.9	60 ± 11	65 ± 8.6	75 ± 8.9
Caffeic acid	102 ± 3.9	96 ± 4.0	43 ± 5.4	78 ± 3.8
Chlorogenic acid	59 ± 16	58 ± 14	13 ± 10	61 ± 13
Cinnamic acid	24 ± 1 1	20 ± 7.3	18 ± 7.5	19 ± 9.8
Eriodictyol	37 ± 6.9	55 ± 4.3	68 ± 10	86 ± 8.0
Ferulic acid	58 ± 8.2	61 ± 5.8	54 ± 12	70 ± 8.9
Gallic acid	54 ± 11	77 ± 15	18 ± 13	1 ± 9.8
Hydroxytyrosol	107 ± 11	95 ± 14	123 ± 10	41 ± 18
Luteolin	67 ± 17	94 ± 22	110 ± 15	92 ± 11
Oleuropein	61 ± 5.1	100 ± 1.5	155 ± 3.2	98 ± 2.1
Pinoresinol	102 ± 13	67 ± 8.9	82 ± 5.4	74 ± 13
Quercetin	26 ± 8.6	53 ± 10	33 ± 5.5	68 ± 3.8
Syringic acid	61 ± 7.3	67 ± 15	40 ± 8.5	56 ± 6.9
Tyrosol	89 ± 5.9	88 ± 8.5	67 ± 6.8	55 ± 7.3
Vanillic acid	60 ± 8.8	102 ± 9.5	53 ± 4.5	80 ± 5.4
Vanillin	22 ± 14	11 ± 18	18 ± 20	18 ± 15
Kaempferol	39 ± 16	47 ± 10	49 ± 18	66 ± 11
Naringenin	40 ± 9.4	37 ± 10	24 ± 13	79 ± 7.8
p-coumaric acid	81 ± 3.1	84 ± 5.6	78 ± 2.8	61 ± 1.6
Rutin	-	88 ± 19	145 ± 22	99 ± 16
Salycilic acid	77 ± 4.9	79 ± 8.8	62 ± 7.9	74 ± 10
Taxifolin	49 ± 5.7	55 ± 7.8	43 ± 3.5	61 ± 10

Table S4. Evaluation of linearity, repeatability, intermediate precision, trueness and selectivity for the determination of phenolic compounds in olive fruits

compound	Linearity R^2%	LOD (mg/kg)	LOQ (mg/kg)	Repeatability RSD_r%			Intermediate precision RSD_R%			Trueness (Accuracy) R%			ME%		
				Level A	Level B	Level C	Level A	Level B	Level C	Level A	Level B	Level C	Level A	Level B	Level C
2 cis-4 trans absicic acid	0.997	0.52	1.5	7.9	17	9.0	9.9	21	8.6	70	102	102	-41	-83	0
2.5 ihydroxybenzoic acid	0.999	0.30	0.91	7.5	7.0	7.0	11	18	8.7	62	87	99	48	49	36
4 hydroxybenzoic acid	1.000	0.15	0.45	13	3.7	3.1	21	8.3	3.8	79	96	93	74	77	76
apigenin	0.983	1.7	5.2	2.7	10	5.3	5.3	15	4.1	78	81	88	0,6	-1,2	-8,2
caffeic acid	0.997	0.55	1.6	11	4.2	7	21	12	6.5	71	86	92			20
chlorogenic acid	0.996	1.4	4.1		17	2.1		33	11						20
cinnamic acid	0.998	0.42	1.3	9.5	14	8.9	8.8	19	8.6	61	85	90	-58	-28	-0,5
citric acid	0.995	0.68	2.0	8,5	11	7,7	13	27	13					42	73
diosmetin	0.948	0.46	1.4	15	14	1.6	15	20	5.3	70	83	97	-23	-18	-9,4
eriodictyol	0.980	1.9	5.7		19	9.4		25	13		136	76			-512
eudesmic acid	0.997	0.50	1.5	8	14	6.9	8.4	17	7.2	75	97	98	79	28	15
ferulic acid	0.997	0.52	1.6	9.2	14	6.4	9.0	18	6.1	68	97	97	-35	8,4	10
homovanillic acid	0.980	3.1	9.2			10			10			98		-11	7,5
hydroxytyrosol	0.985	1.6	4.9	3.4	13	11	6.0	12	8.3	98	100	95		-285	-2,7
naringenin	0.978	1.4	4.3	14	11	7.0	11	14	5.9	67	93	98	0,86	66	7,4
oleuropein	0.997	0.57	1.7	7.4	15	8.9	7.0	19	7.8	82	111	98	-327	-348	-73
p coumaric acid	0.985	1.2	3.6	8.9	4.1	7.2	10	12	7.2	75	93	105	6,7	7,7	6,4
pinoresinol	0.998	0.47	1.4	8.5	16	6.8	9.8	21	7.5	76	109	100	-79	-44	-13
quercetin	0.983	1.3	3.9	23	5.0	20	20	22	15	59	60	66			
quinic acid	0.994	1.7	5.1			14			11			77			97
rutin	0.989	2.3	6.9		22	19		32	17		140	93			-243
salicylic acid	0.985	1.2	3.6	7.7	9.8	6.5	8.5	14	7	72	89	90	7,1	-25	6,4
sinapic acid	0.995	0.66	2.0	10	10	7.6	9.1	18	9.6	62	96	96		-1,6	1,8
syringic acid	0.997	1.2	3.7		7.1	3.4		10	6.7		107	90		16	43
tyrosol	0.952	3.6	11	2.5	15	11	5.0	12	8.6	95	100	94	155	-189	-29
vanillic acd	0.999	0.39	1.2	11	8.2	2.8	11	10	4.2	106	98	98		51	66
vanillin	0.997	0.55	1.7	28	11	25	21	13	20	25	47	61	-248	-83	-8,7

Table S5. Target list of phenolic compounds

Compound	Molecular formula	$[M-H]^-$ m/z	tR (min)	q1 m/z	q1 formula	q2 m/z	q2 formula	q3 m/z	q3 formula	q4 m/z	q4 formula
Ferulic acid	C10H10O4	193.050632	3	134.037	C8H6O2	178.0271	C9H6O4				
Apigenin	C15H10O5	269.045547	8.24	269.0455	C15H9O5	117.0346	C8H5O	151.0037	C7H3O4	65.0033	C4HO
Luteolin	C15H10O6	285.040462	7.55	285.0405	C15H9O6	133.0295	C8H5O2				
kaempferol	C15H10O6	285.040462	8	229.0502	C13H9O4	185.061	C12H9O2	133.0297	C8H5O2		
Quercetin	C15H10O7	301.035376	7.2	151.0025	C7H3O4	178.9975	C8H3O5	121.0284	C7H5O2		
naringenin	C15H12O5	271.061197	7.2	119.0491	C8H7O	151.0025	C7H3O4	177.0182	C9H5O4		
Eriodictyol	C15H12O6	287.056112	6.4	151.0025	C7H3O4	135.044	C8H7O2				
Catechin	C15H14O6	289.071762	3.87	203.0705	C12H11O3	123.0458	C7H7O2				
Chlorogenic acid	C16H18O9	353.087806	2.7	191.0566	C7H11O6						
oleocanthal	C17H20O5	303.123797	6.6	137.0611	C8H9O2	165.0552	C9H9O3	183.0666	C9H11O4	95.0492	C6H7O
oleacin	C17H20O6	319.118712	5.8	69.0346	C4H5O	95.0502	C6H7O	70.0424	C4H6O	139.0765	C8H11O2
oleocanthalic acid	C17H20O6	319.118712	5.2	199.0615	C9H11O5	111.0085	C5H3O3				
Lingstroside aglycone	C19H22O7	361.129277	8.3	259.0975	C15H15O4	291.0875	C15H15O6	127.0396	C6H7O3		
Oleokoronal	C19H22O7	361.129277	6.8	259.0975	C15H15O4	291.0875	C15H15O6	171.0279	C7H7O5		
oleomissional	C19H22O8	377.124191	6	275.0555	C15H15O5	307.0823	C15H15O7	139.0398	C7H7O3	149.0242	C8H5O3
Oleuropein aglycone	C19H22O8	377.124191	7.29	111.0088	C5H3O3	149.0244	C8H5O3	195.0644	C10H11O4	275.0919	C15H15O5
Pinoresinol	C20H22O6	357.134362	6.49	151.0399	C8H7O3	136.0163					
Oleuropein	C25H32O13	539.177015	5.96	121.0295	C7H5O2	307.0823	C15H15O7	377.1242	C19H21O8	275.0924	C15H15O5
salycilic acid	C7H6O3	137.024418	3.7	93.0346	C6H5O	65.0397	C5H5				
Gallic acid	C7H6O5	169.014247	1.25	125.0244	C6H5O3	69.0346	C4H5O				
Tyrosol	C8H10O2	137.060803	4.07	93.0345	C6H5O	119.0506	C8H7O				
Hydroxytyrosol	C8H10O3	153.055718	3.53	123.0446	C7H7O2						
Vanillin	C8H8O3	151.040068	4.73	71.014	C3H3O2	95.014	C5H3O2	108.0217	C6H4O2	136.0162	C7H4O3
Vanillic acid	C8H8O4	167.034982	1.6	152.0109	C5H2N3O3	125.0233	C6H5O3	91.0177	C6H3O	108.0212	
Homovanillic acid	C9H10O4	181.050632	2.4	69.0352	C4H5O	122.0369	C7H6O2	134.0361		C8H7O2^1 -	135.0445
Syringic acid	C9H10O5	C9H10O5	2	123.0076	C6H3O3	166.9975	C7H3O5				
Cinnamic acid	C9H8O2	147.045153	4.5	147.044	C9H7O2						
p-coumaric acid	C9H8O3	163.040068	2.6	119.0506	C8H7O	93.0349	C6H5O				
Caffeic acid	C9H8O4	179.034982	1.9	135.044	C8H7O2	134.0348	C6H4N3O				
taxifolin	C15H12O7	303.051026	4.9	285.0393	C15H9O6	125.0233	C6H5O3	153.0182	C7H5O4		
rutin	C27H30O16	609.146108	5.5	301.0335	C15H9O7	146.963					
verbascoside	C29H36O15	623.198144	4.9	161.0242	C9H5O3	303.554	C8H15O12	461.1652	C20H29O12		
2.5dihydroxybenzoic acid	C7H6O4	153.019332	2.41	108.0217	C6H4O2	109.0295	C6H5O2	81.0346	C5H5O		
2-cis.4-trans-Abscisic acid	C15H20O4	263.128883	5.02	204.1156	C13H16O2	219.1391	C14H19O2	151.0765	C9H11O2		
3.4-Dihydroxybenzoic acid	C7H6O4	153.019332	1.35	109.0295	C6H5O2	108.0217	C6H4O2	81.0346	C5H5O		
4-hydroxybenzoic acid	C7H6O3	137.024418	1.45	93.0346	C6H5O	65.0397	C5H5				
diosmetin	C16H12O6	299.056112	8.2	284.0332	C13H6N3O5	151.0025	C7H3O4				
sinapic acid	C11H12O5	223.061197	3.1	193.0145	C9H5O5	149.0244	C8H5O3	121.0307	C7H5O2	135.0445	C8H7O2^1-
quinic acid	C7H12O6	191.056112	1.2	127.0398	C7H7O3	171.0299	C7H7O5	111.012	C5H3O3		
eudesmic acid	C10H12O5	211.061197	3.4	137.0244	C7H5O3	167.0699	C9H11O3	152.0471	C11H6N		
citric acid	C6H8O7	191.019726	1.1	111.0062	C5H3O3	129.0205	C6HN4				
neochlorogenic acid	C16H18O9	353.087806	2.8	191.0555	C7H11O6	179.0347	C9H7O4				

Table S6. Suspect list of bioactive compounds encountered in olive samples

ID	Compound name	Molecular formula	monoisotopic mass	[M-H]-	Rt predicted	Qual.1	Qual.2	Qual.3	Qual.4	Reference
1	3.4-Dihydroxyphenylglycol	$C_8H_{10}O_4$	170.0579	169.0506	4.52	123.0430	122.0347	151.0409	-	[7]
2	4-O-methyl-D-glucuronic acid	$C_7H_{12}O_7$	208.0583	207.0510	2.14	-	-	-	-	[71]
3	Vicenin 2	$C_{27}H_{30}O_{15}$	594.1585	593.1512	4.35	353.0645	383.0761	473.1053	-	[68]
4	Scolymoside (luteolin 7-rutinoside)	$C_{27}H_{30}O_{15}$	594.1585	593.1512	6.98	-	-	-	-	[70]
5	Quercetin 3-O-glucoside	$C_{21}H_{20}O_{12}$	464.0955	463.0882	5.91	300.0257	301.0313	271.0222		[68]
6	Chrysoeriol-7-O-glucoside	$C_{22}H_{22}O_{11}$	462.1162	461.1089	7.09	-	-	-	-	[13]
7	luteolin -4 glucoside	$C_{21}H_{20}O_{11}$	448.1011	447.0933	6.54	285.0384	284.0320	286.0395	-	[59]
8	luteolin -8 glucoside	$C_{21}H_{20}O_{11}$	448.1011	447.0933	4.79	285.0384	284.0320	286.0395	-	[59]
9	luteolin -3 glucoside	$C_{21}H_{20}O_{12}$	448.1011	447.0933	6.58	285.0384	284.0320	286.0395	-	[59]
10	Berchemol	$C_{20}H_{24}O_7$	376.1522	375.1449	8.73	-	-	-	-	[71]
11	Oleuropein diglucoside	$C_{31}H_{42}O_{18}$	702.2371	701.2298	-	341.0000	539.0000	377.0000	307.0000	[16]
12	nuzhenide	$C_{31}H_{42}O_{17}$	686.2422	685.2349	9.4	453.1389	421.1495	299.1130	523.1809	[58]
13	dihydrooleuropein	$C_{25}H_{36}O_{13}$	544.2155	543.2083	-	525.1980	513.1982	-	-	[42]
14	Hydroxytyrosol acyclodihydroelenolate	$C_{19}H_{26}O_8$	382.1628	381.1555	-	363.1450	349.1289	331.1185	213.0761	[42]
15	Isoverbascoside	$C_{29}H_{36}O_{15}$	624.2054	623.1981	8.49	461.1659	161.0242	-	-	[58]
16	Caffeoyl-6'-secologanoside	$C_{25}H_{28}O_{14}$	552.1479	551.1406	-	-	-	-	-	[42]
17	Comselogoside	$C_{25}H_{28}O_{13}$	536.1530	535.1457	-	491.1560	265.0728	-	-	[42]
18	Hydroxytyrosol-1-β-glucoside	$C_{14}H_{20}O_8$	316.1158	315.1085	4.95	153.0557	123.0451	-	-	[45]
19	Hydroxytyrosol-3-β-glucoside	$C_{14}H_{20}O_8$	316.1158	315.1085	5	153.0557	123.0451	-	-	[45]
20	tyrosol acetate	$C_{10}H_{12}O_3$	180.0786	179.0714	-	-	-	-	-	[58]
21	3'-Hydroxybiphenyl-4-carboxylic acid	$C_{13}H_{10}O_3$	214.0630	213.0557	4.1	-	-	-	-	[59]
22	methyl gallate	$C_8H_8O_5$	184.0372	183.0299	4.59	168.0045	124.0163	-	-	[69]
23	ellagic acid	$C_{14}H_6O_8$	302.0063	300.9990	4.87	257.0084	229.0144	-	-	[69]
24	Brevifolincarboxylic acid	$C_{13}H_8O_8$	292.0219	291.0146	2.41	-	-	-	-	[69]
25	Delphinidin 3-glucoside	$C_{21}H_{21}O_{12+}$	465.1028	464.0960	6.69	-	-	-	-	[4]
26	ethyl gallate	$C_9H_{10}O_5$	198.0528	197.0455	5.14	-	-	-	-	[69]
27	kaempferol-7-O-hexoside	$C_{21}H_{20}O_{11}$	448.1006	447.0933	6.25	285.0393	284.0316	286.0423	-	[69]
28	Galactosylglycerol	$C_9H_{18}O_8$	254.1002	253.0929	3.27	-	-	-	-	[72]
29	2.4.5-trihydroxypentanoic acid	$C_5H_{10}O_5$	150.0528	149.0455	1.77	-	-	-	-	[72]
30	glucaric acid	$C_6H_{10}O_8$	210.0376	209.0303	1.64	-	-	-	-	[72]
31	ribonic acid	$C_5H_{10}O_6$	166.0477	165.0405	1.48	-	-	-	-	[72]
32	heptanoic acid	$C_7H_{14}O_2$	130.0994	129.0921	5.9	-	-	-	-	[72]
33	malonic acid	$C_3H_4O_4$	104.0110	103.0037	0.98	59.0136	-	-	-	[72]
34	fumaric acid	$C_4H_4O_4$	116.0110	115.0037	2.06	71.0123	-	-	-	[72]
35	elenolic acid methyl ester	$C_{12}H_{16}O_6$	256.0947	255.0874	-	-	-	-	-	[13]

36	gallocatechin	$C_{15}H_{14}O_7$	306.0740	305.0667	6.69	125.0249	137.0248	109.0295	-	[13]
37	Aromadedrin	$C_{15}H_{12}O_6$	288.0634	287.0561	7.32	-	-	-	-	[13]
38	acyclodihydroelenolic acid hexoside	$C_{17}H_{28}O_{11}$	408.1632	407.1559	-	-	-	-	-	[13]
39	decarboxymethylelenolic acid	$C_9H_{12}O_4$	184.0736	183.0663	-	-	-	-	-	[13]
40	2-phenethyl β-primeveroside	$C_{19}H_{28}O_{10}$	416.1682	415.1610	5.19	-	-	-	-	[13]
41	desoxyelenolic acid	$C_{11}H_{14}O_5$	226.0841	225.0768	-	-	-	-	-	[13]
42	azelaic acid	$C_9H_{16}O_4$	188.1049	187.0976	3.63	-	-	-	-	[13]
43	acetylated hydroxytyrosol	$C_{10}H_{12}O_4$	196.0736	195.0663	-	-	-	-	-	[13]
44	Lucidumoside C	$C_{27}H_{36}O_{14}$	584.2105	583.2032	10.74	-	-	-	-	[13]
45	trihydroxyoctadecenoic acid	$C_{18}H_{34}O_5$	330.2406	329.2333	7.03	-	-	-	-	[13]
46	trihydroxyoctadecanoic acid	$C_{18}H_{36}O_5$	332.2563	331.2490	11.59	-	-	-	-	[13]
47	methyldecarboxymethyloleuropein aglycone	$C_{18}H_{22}O_6$	334.1416	333.1344	-	-	-	-	-	[13]
48	gingerol	$C_{17}H_{26}O_4$	294.1831	293.1758	10.28	193.0822	99.0798	57.0294	-	[13]
49	hydroxyoctadecatrienoic acid	$C_{18}H_{30}O_3$	294.2195	293.2122	10.25	-	-	-	-	[13]
50	dihydroxyoctadecanoic acid	$C_{18}H_{36}O_4$	316.2614	315.2541	12.55	-	-	-	-	[13]
51	hydroxyoctadecadienoic acid	$C_{18}H_{31}O_3$	295.2273	294.2200	10.34	-	-	-	-	[13]
52	dihydroxyoctadecadienoic acid	$C_{18}H_{32}O_4$	312.2301	311.2228	-	-	-	-	-	[13]
53	hydroxyoctadecenoic acid	$C_{18}H_{34}O_3$	298.2508	297.2435	10.39	-	-	-	-	[13]
54	hydroxyoctadecanoic acid	$C_{18}H_{36}O_3$	300.2664	299.2592	13.07	-	-	-	-	[13]
55	fustin	$C_{15}H_{12}O_6$	288.0634	287.0561	6.86	-	-	-	-	[47]
56	Chrysoeriol	$C_{16}H_{12}O_6$	300.0634	299.0561	8.02	284.0300	285.0330	-	-	[58]
57	caffeoylquinic acid	$C_{16}H_{18}O_9$	354.0951	353.0878	3.7	191.0353	179.0331	-	-	[20]
58	phloretic acid	$C_9H_{10}O_3$	166.0630	165.0557	4.88	106.0358	119.0433	101.0310	91.0483	[1]
59	3-(3.4-Dihydroxyphenyl)propanoic acid	$C_9H_{10}O_4$	182.0579	181.0506	-	-	-	-	-	[1]
60	p-Hydroxyphenylacetic acid	$C_8H_8O_3$	152.0473	151.0401	3.97	107.0507	107.0628	151.0414	79.0549	[1]
61	3.4-Dihydroxyphenylacetic acid	$C_8H_8O_4$	168.0423	167.0350	-	121.0259	59.9842	59.9972	-	[1]
62	Demethyloleuropein	$C_{24}H_{30}O_{13}$	526.1686	525.1614	7.37	-	-	-	-	[10]
63	dimethyloleuropein aglycone	$C_{21}H_{26}O_8$	406.1628	405.1555	5.8	-	-	-	--	[13]
64	salidroside	$C_{14}H_{20}O_7$	300.1209	299.1136	4.85	89.0237	59.0141	71.0134	119.0477	[10]
65	cyanidin-3-O-b-D-glucoside	$C_{21}H_{22}O_{11}$	450.1162	449.1089	7.05	284.0333	285.0389	256.0362		[59]
66	Cyanidin 3-rutinoside	$C_{27}H_{32}O_{15}$	596.1741	595.1668	7.26	284.0319	285.0360	285.0490	-	[59]
67	Luteolin 7-glucoside	$C_{21}H_{20}O_{11}$	448.1006	447.0933	6.61	285.0384	284.0320	286.0395	-	[7]
68	Hydroxytyrosol 4'-O-glucoside	$C_{14}H_{20}O_8$	316.1158	315.1085	5.51	153.0557	-	-	-	[10]
69	Quercitrin	$C_{21}H_{20}O_{11}$	448.1006	447.0933	7.03	300.0278	301.0345	271.0250	-	[8]
70	Apigenin 7-glucoside	$C_{21}H_{20}O_{10}$	432.1056	431.0984	6.39	268.0356	269.0418	-	-	[10]
71	oleuroside	$C_{25}H_{32}O_{13}$	540.1843	539.1770	9.35	-	-	-	-	[47]
72	b-tocotrienol	$C_{26}H_{38}O_2$	382.2872	381.2799	13.19	-	-	-	-	[9]
73	a tocopherol	$C_{29}H_{50}O_2$	430.3811	429.3738	12.61	-	-	-	-	[9]
74	g tocopherol	$C_{29}H_{48}O_2$	428.3654	427.3582	12.74	-	-	-	-	[9]

75	retinol	$C_{20}H_{30}O$	286.2297	285.2224	11.81	-	-	-	-	[9]
76	thiamine	$C_{12}H_{17}N_4OS$	401.0978	400.0905	-	147.0690	148.0706	234.0926	-	[9]
77	rivoflavin	$C_{17}H_{20}N_4O_6$	376.1383	375.1310	-	255.0938	375.1325	-	-	[9]
78	ascorbic acid	$C_6H_8O_6$	176.0321	175.0248	-	87.0078	-	-	-	[9]
79	b carotene	$C_{40}H_{56}$	536.4382	535.4309	-	-	-	-	-	[9]
80	Isorhoifolin	$C_{40}H_{56}O_3$	584.4229	583.4157	6.81	577.1702	269.0475	270.0497	-	[10]
81	Peonidin 3-glucoside	$C_{22}H_{23}ClO_{11}$	498.0929	497.0856	7.6	299.0539	283.0263	298.0415	-	[10]
82	Delphinidin-3-rhamnoglucoside	$C_{27}H_{31}ClO_{15}$	630.1351	629.1279	5.6	-	-	-	-	[10]
83	Hexahydroxydiphenoyl hexosyl-gallate	$C_{27}H_{22}O_{18}$	634.0806	633.0733	-	463.0504	301.9971	-	-	[12]
84	hydrooleuropein	$C_{30}H_{22}O_{10}$	542.1213	541.1140	-	415.0819	389.1018	-	-	[12]
85	oleoside 11 methyl ester	$C_{17}H_{24}O_{11}$	404.1319	403.1246	4.85	223.0612	-	-	-	[19]
86	succinic acid	$C_4H_6O_4$	118.0266	117.0193	1.29	73.0130	-	-	-	[19]
87	oxalic acid	$C_2H_2O_4$	89.9953	88.9880	0.83	-	-	-	-	[72]
88	diosmetin -7 glucoside	$C_{22}H_{22}O_{11}$	462.1162	461.1089	7.02	-	-	-	-	[2]
89	oleoside	$C_{16}H_{22}O_{11}$	390.1162	389.1089	5.8	345.1176	209.0460	165.0558	183.0666	[57]
90	glucosyl methyl oleoside	$C_{23}H_{34}O_{16}$	566.1847	565.1774	-	-	-	-	-	[57]
91	Aesculetin	$C_9H_6O_4$	178.0266	177.0193	3.93	177.0206	133.0298	105.0349	-	[13]
92	Aesculin	$C_{15}H_{16}O_9$	340.0794	339.0722	3.42	177.0197	-	-	-	[13]
93	dehydrooleuropein aglycone	$C_{19}H_{20}O_8$	376.1158	375.1085	-	-	-	-	-	[13]
94	hydroxyelenolic acid	$C_{11}H_{14}O_7$	258.0740	257.0667	-	-	-	-	-	[13]
95	erythrodiol	$C_{30}H_{50}O_2$	442.3811	441.3738	12.84	-	-	-	-	[13]
96	uvaol	$C_{30}H_{50}O_2$	442.3811	441.3738	12.83	-	-	-	-	[13]
97	octadecanoic acid	$C_{18}H_{36}O_2$	284.2715	283.2643	13.07	-	-	-	-	[13]
98	oleic acid	$C_{18}H_{34}O_2$	282.2559	281.2486	13.28	-	-	-	-	[13]
99	linoleic acid	$C_{18}H_{32}O_2$	280.2402	279.2330	12.92	-	-	-	-	[13]
100	linolenic acid	$C_{18}H_{30}O_2$	278.2246	277.2173	12.37	-	-	-	-	[13]
101	palmitic acid	$C_{16}H_{32}O_2$	256.2402	255.2330	12.94	-	-	-	-	[13]
102	palmitoleic acid	$C_{16}H_{30}O_2$	254.2246	253.2173	12.44	217.8511	181.1071	-	-	[13]
103	10 hydroxydecanoic acid	$C_{10}H_{20}O_3$	188.1412	187.1340	6.49	141.1290	169.1254	-	-	[13]
104	9-Hydroxy-10.12.15-octadecatrienoic acid	$C_{18}H_{30}O_3$	294.2195	293.2122	-	-	-	-	-	[13]
105	(S)-10.16-Dihydroxyhexadecanoic acid	$C_{16}H_{32}O_4$	288.2301	287.2228	-	-	-	-	-	[13]
106	18-trihydroxyoctadecadienoic acid	$C_{18}H_{32}O_5$	328.2250	327.2177	9.05	-	-	-	-	[13]
107	9.10.13-Trihydroxy-11-octadecenoic acid	$C_{18}H_{34}O_5$	330.2406	329.2333	-	-	-	-	-	[13]
108	methoxynuzhenide	$C_{32}H_{44}O_{18}$	716.2528	715.2455	-	-	-	-	-	[16]
109	methoxynuzhenide 11 methyl oleoside	$C_{49}H_{66}O_{28}$	1102.3741	1101.3668	-	715.2449	553.1921	329.1236	-	[16]
110	methoxyoleuropein	$C_{26}H_{34}O_{14}$	570.1949	569.1876	-	-	-	-	-	[47]
111	Forsythiaside	$C_{29}H_{36}O_{15}$	624.2054	623.1981	8.45	-	-	-	-	[47]
112	Homovanillyl alcohol	$C_9H_{12}O_3$	168.0786	167.0714	6.29	-	-	-	-	[47]

113	Dimeresculetin	$C_8H_{10}O_8$	234.0376	233.0303	-	-	-	-	-	[47]
114	scopoletin	$C_{10}H_8O_4$	192.0423	191.0350	4.84	176.0112	177.0153	148.0166	-	[47]
115	Naringenin 4'-O-glucoside	$C_{21}H_{22}O_{10}$	434.1213	433.1140	5.95	271.0573	150.9990	119.0464	-	[47]
116	hesperidin	$C_{28}H_{34}O_{15}$	610.1898	609.1825	-	301.0718	-	-	-	[47]
117	cyanidin	$C_{15}H_{11}O_6$	287.0556	286.0483	-	183.0410	159.0414	211.0360	239.0319	[58]
118	cyanidin chloride	$C_{15}H_{11}ClO_6$	322.0244	321.0171	7.72	-	-	-	-	[43]
119	cyanidin 3 glucoside chloride	$C_{21}H_{21}ClO_{11}$	484.0772	483.0700	7.05	-	-	-	-	[43]
120	Isoorientin	$C_{21}H_{20}O_{11}$	448.1006	447.0933	5.07	357.0615	358.0641		-	[43]
121	hydroxyeicosanoic acid	$C_{20}H_{40}O_3$	328.2977	327.2905	13.86	-	-	-	-	[13]
122	ursolic acid	$C_{30}H_{48}O_3$	456.3603	455.3531	12.31	-	-	-	-	[13]
123	hydroxytyrosol acetate	$C_{10}H_{12}O_4$	196.0736	195.0663	6.44	-	-	-	-	[58]
124	elenolic acid	$C_{11}H_{14}O_6$	242.0790	241.0718	3.4	95.0496	127.0400	151.0402	171.0300	[13]
125	Hydroxylated form of elenolic acid	$C_{11}H_{14}O_7$	258.0740	257.0667	-	137.0603	181.0535	-	-	[42]
126	10-Hydroxy decarboxymethyl oleuropein aglycone	$C_{17}H_{20}O_7$	336.1209	335.1136	-	85.0296	121.0292	151.0401	153.0557	[13]
127	10-hydroxy oleuropein aglycone	$C_{19}H_{22}O_9$	394.1264	393.1191	-	137.0244	181.0502	-	-	[13]
128	Methyl oleuropein aglycone	$C_{20}H_{24}O_8$	392.1471	391.1398	-	111.0087	137.0608	291.0875	67.0192	[13]
129	1-Acetoxypinoresinol	$C_{22}H_{24}O_8$	416.1471	415.1398	8.18	151.0402	280.0951	343.1188	-	[13]
130	Syringaresinol	$C_{22}H_{26}O_8$	418.1628	417.1555	9.14	127.0408	181.0506	-	-	[13]
131	1-Hydroxypinoresinol	$C_{20}H_{22}O_7$	374.1366	373.1293	7.38	121.0294	151.0401	163.0402	-	[13]

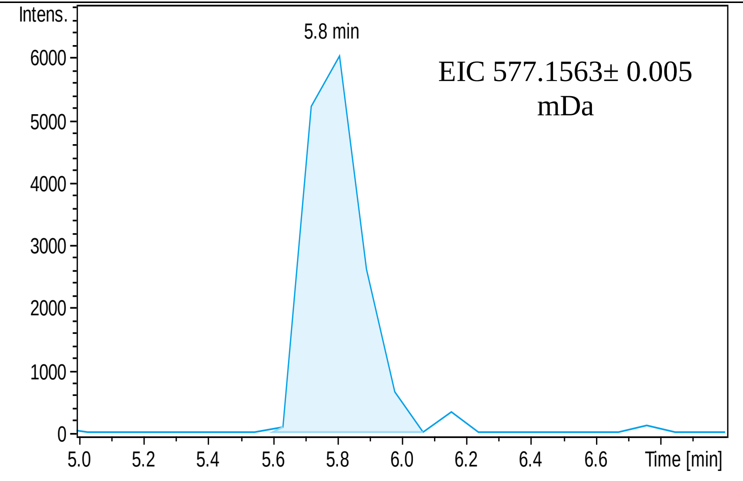

Fig S1a. EIC of m/z 577.1563 in an edible olive fruit

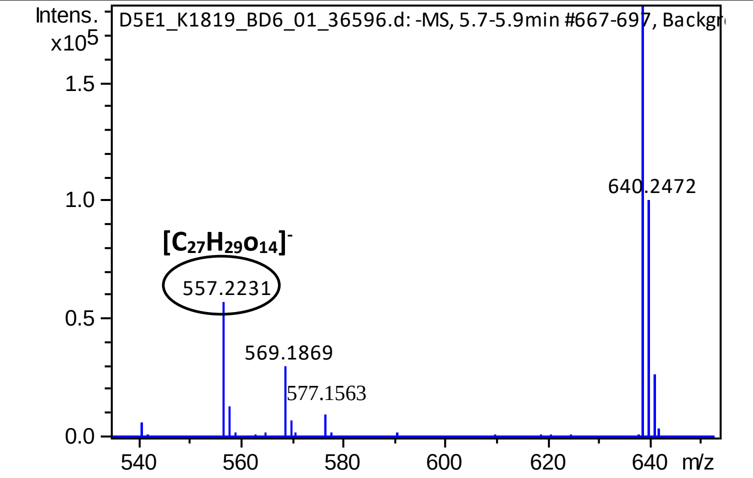

Fig S1b. Background subtracted MS Spectra from 5.7 to 5.8

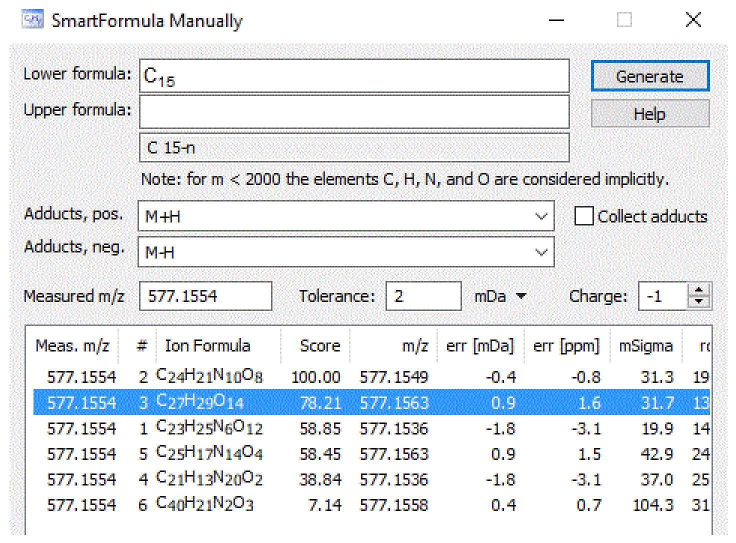

Meas. m/z	#	Ion Formula	Score	m/z	err [mDa]	err [ppm]	mSigma	r[illegible]
577.1554	2	$C_{24}H_{21}N_{10}O_8$	100.00	577.1549	-0.4	-0.8	31.3	19
577.1554	3	$C_{27}H_{29}O_{14}$	78.21	577.1563	0.9	1.6	31.7	13
577.1554	1	$C_{23}H_{25}N_6O_{12}$	58.85	577.1536	-1.8	-3.1	19.9	14
577.1554	5	$C_{25}H_{17}N_{14}O_4$	58.45	577.1563	0.9	1.5	42.9	24
577.1554	4	$C_{21}H_{13}N_{20}O_2$	38.84	577.1536	-1.8	-3.1	37.0	25
577.1554	6	$C_{40}H_{21}N_2O_3$	7.14	577.1558	0.4	0.7	104.3	31

Fig S1c. Molecular Formula Annotation of m/z 593.1512

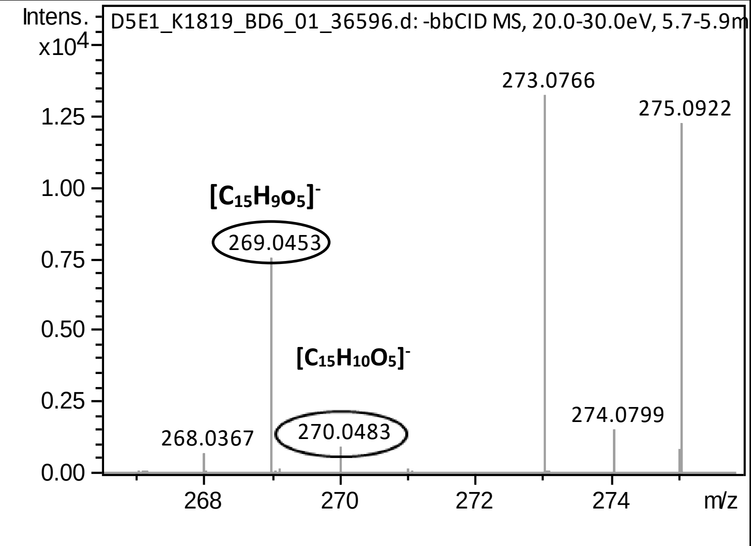

Fig S1d. Background subtracted MS/MS Spectra from 5.7 to 5.8

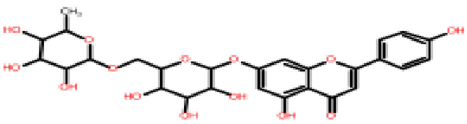

$[C_{27}H_{30}O_{14}]$

Isorhoifolin

Precursor ion

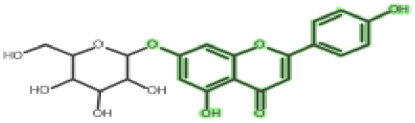

$[C_{15}H_9O_5]^-$

Mass error:0.9

Fragment ion

Fig S1e. Structures of precursor and fragment ions of isorhoifolin

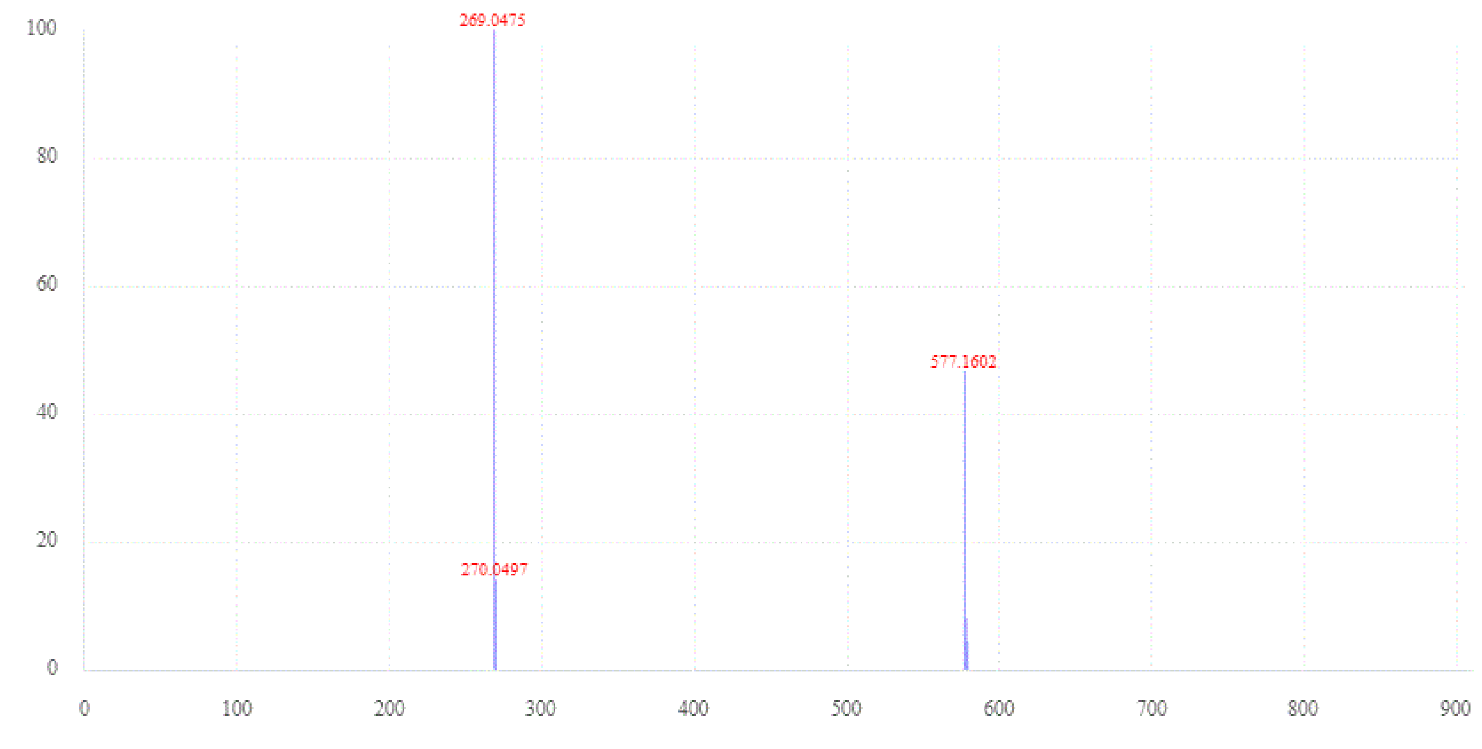

Fig S1f. Bruker Sumner MetaboBASE Plant Library Spectrum MetaboBASE0225 (Isorhoifolin)

Figure S1. Identification data for the mass feature m/z 577.1602_5.7 min (Isorhoifolin)

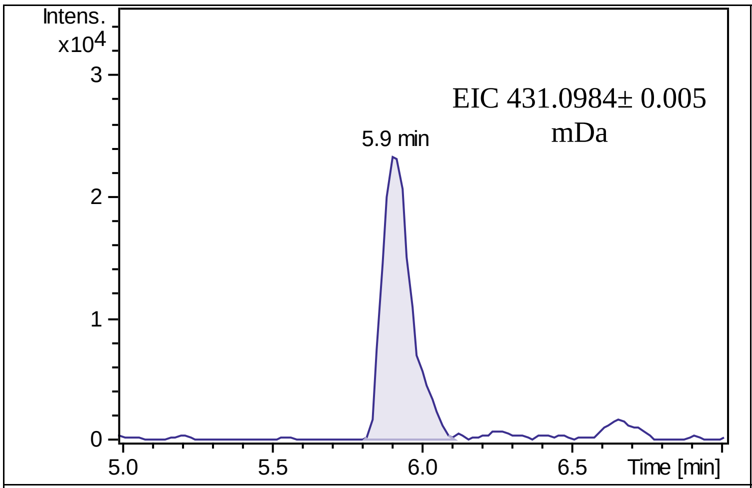

Fig S2a. EIC of m/z 431.0984 in an edible olive fruit

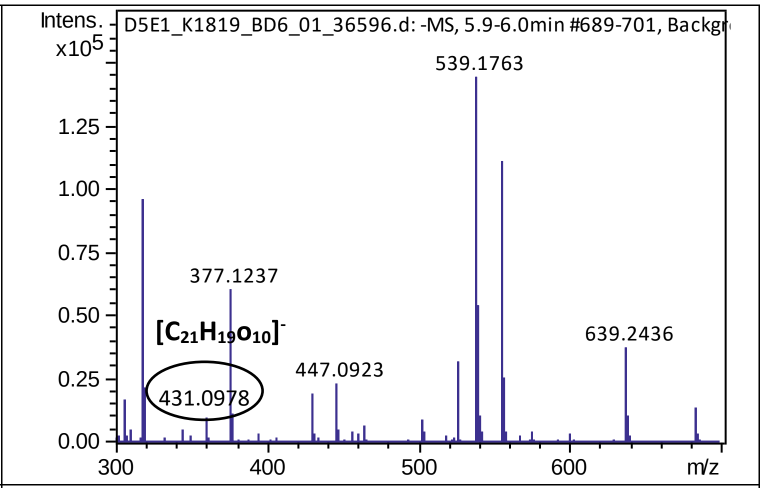

Fig S2b. Background subtracted MS Spectra from 5.9 to 6

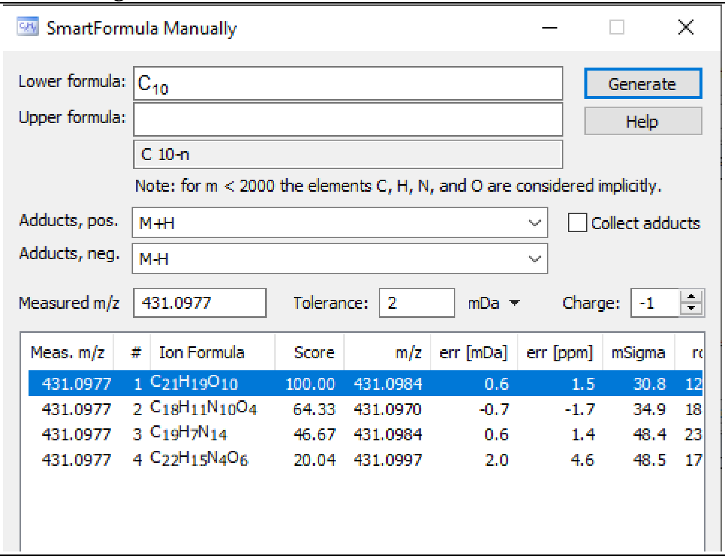

Fig S2c. Molecular Formula Annotation of m/z 593.1512

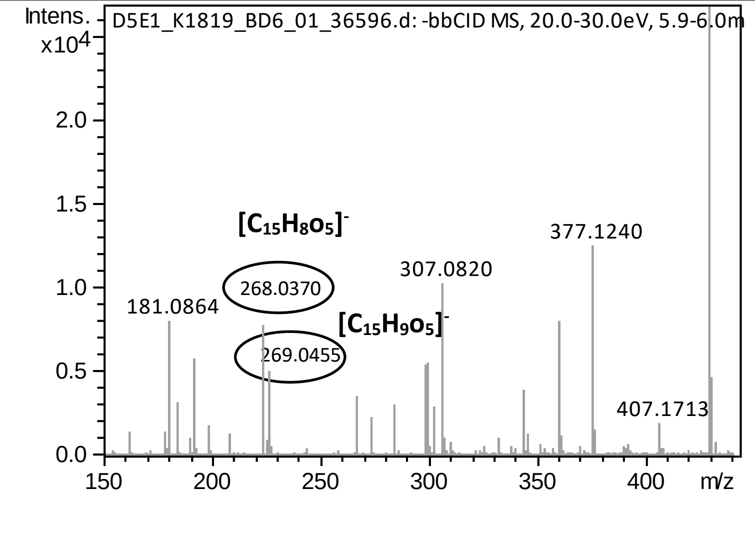

Fig S2d. Background subtracted MS/MS Spectra from 5.9 to 6

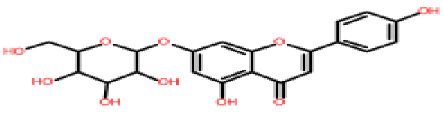

[C21H20O10]
Apigenin-7 glucoside
Precursor ion

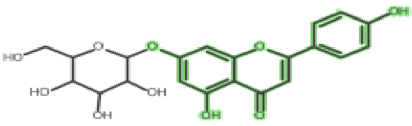

[C15H9O5]-
Mass error:0.7 mda

Fragment ion

Fig S2e. Structures of precursor and fragment ions of Apigenin-7 glucoside

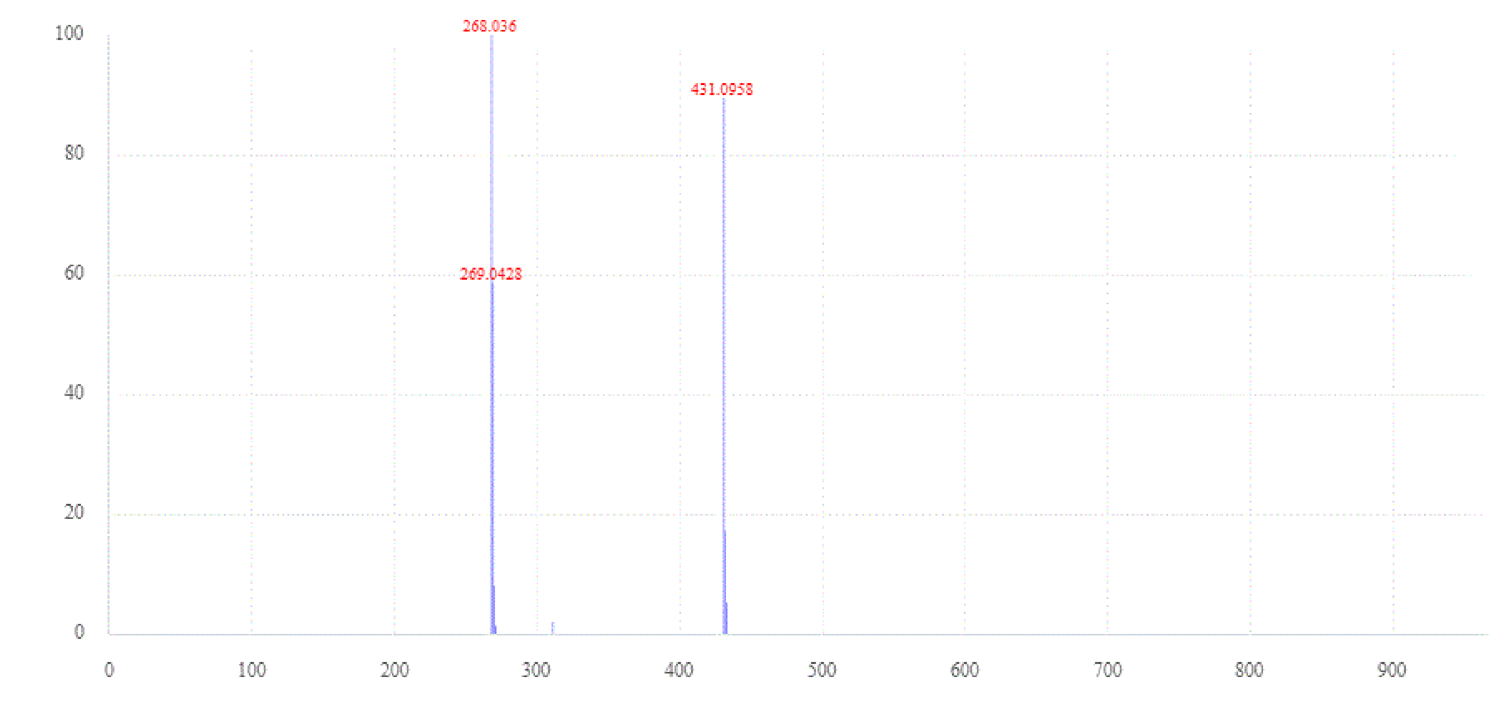

Fig S2f. Bruker Sumner MetaboBASE Plant Library Spectrum MetaboBASE0712 (apigenin-7 glucoside)

Figure S2. Identification data for the mass feature m/z 431.0984_5.9 min (apigenin-7 glucoside)

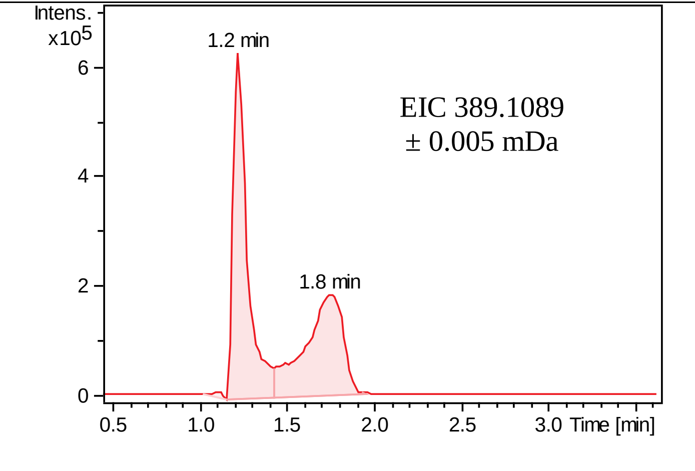

Fig S3a. EIC of m/z 389.1089 in an edible olive fruit

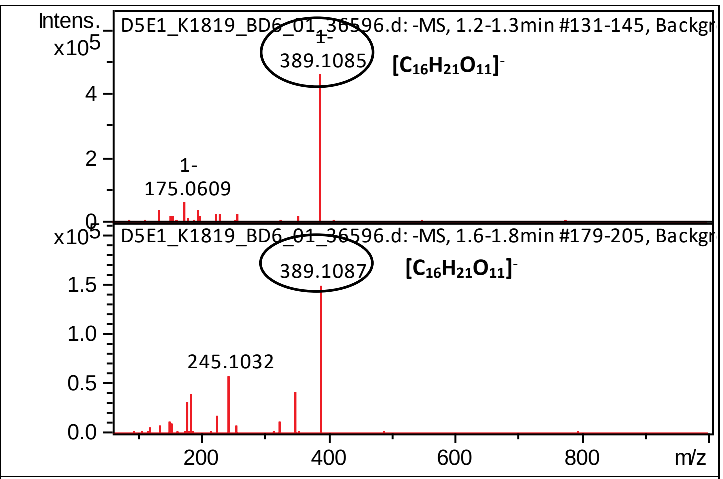

Fig S3b. Background subtracted MS Spectras from 1.2 to 1.3 min and from 1.6 to 1.8 min

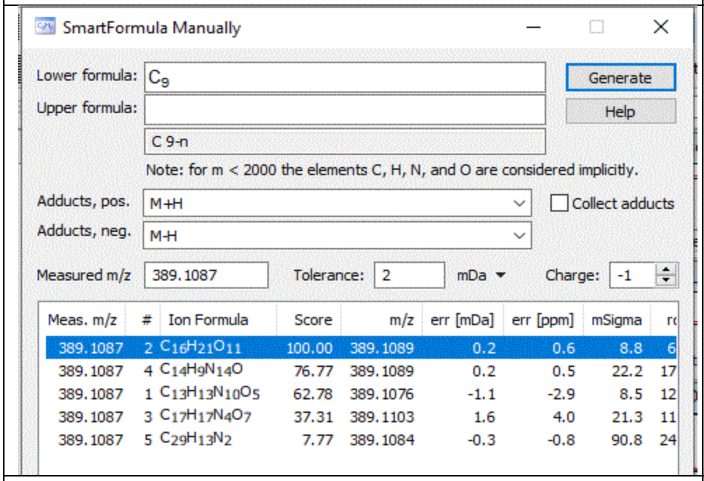

Fig S3c. Molecular Formula Annotation of m/z 389.1089

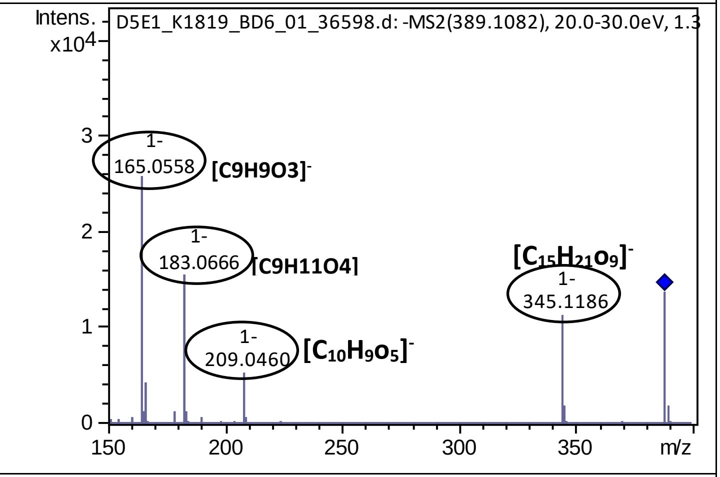

Fig S3d. Background subtracted MS/MS Spectra from 1.2 to 1.3 min

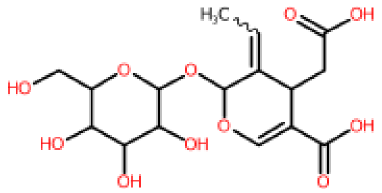

[$C_{16}H_{22}O_{11}$]

oleoside

Precursor ion

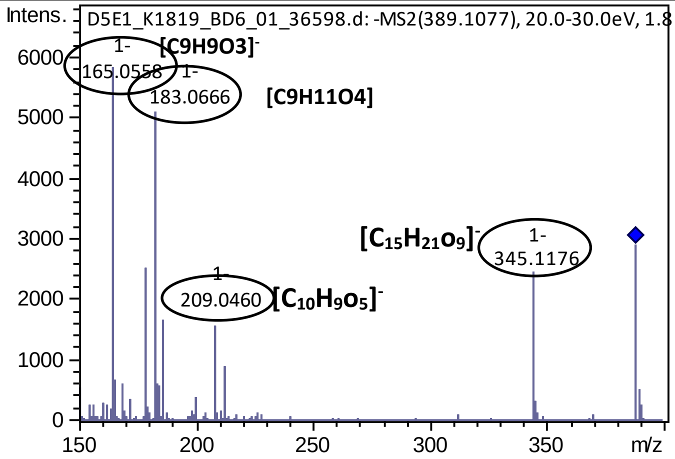

Fig S3e. Structures of precursor

Fig S3f. Background subtracted MS/MS Spectra from 1.6 to 1.8 min

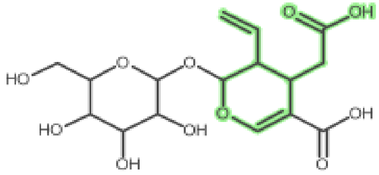

[$C_9H_9O_3$]$^-$
Mass error: 0.1 mDa

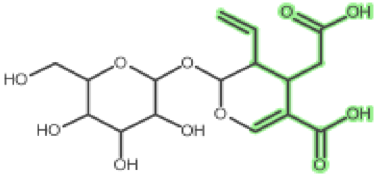

[$C_9H_{11}O_4$]$^-$
Mass error: 0.3 mDa

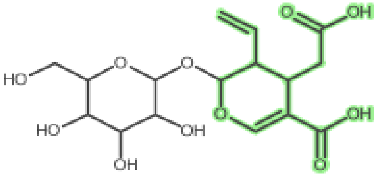

[$C_{10}H_9O_5$]$^-$
Mass error: 0.5 mDa

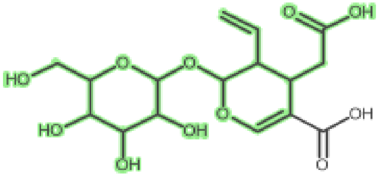

[$C_{15}H_{21}O_9$]$^-$
Mass error: 0.5 mDa

Fig S3g. Structures of fragment ions of Oleoside

Figure S3. Identification data for the mass feature m/z 389.1089_1.8 min (oleoside)

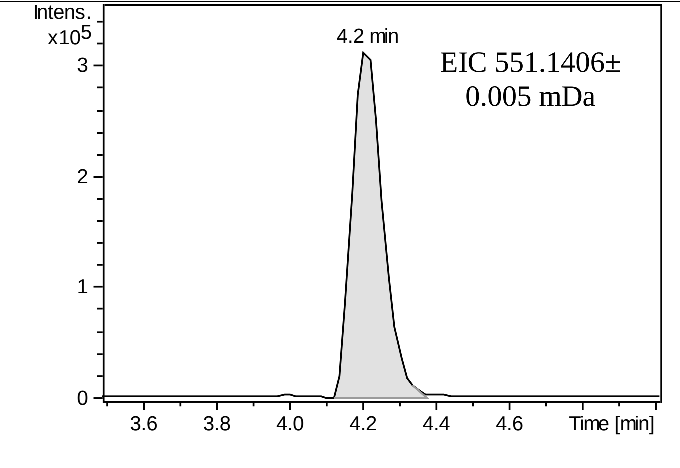

Fig S4a. EIC of m/z 447.0933 in an edible olive fruit

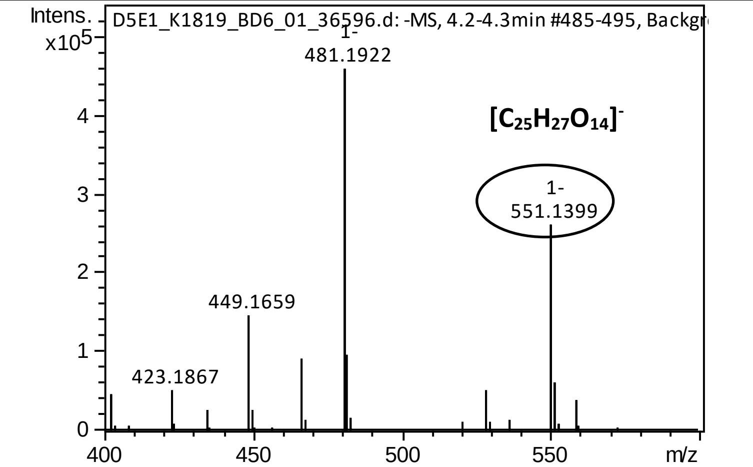

Fig S4b. Background subtracted MS Spectra from 4.2 to 4.3 min

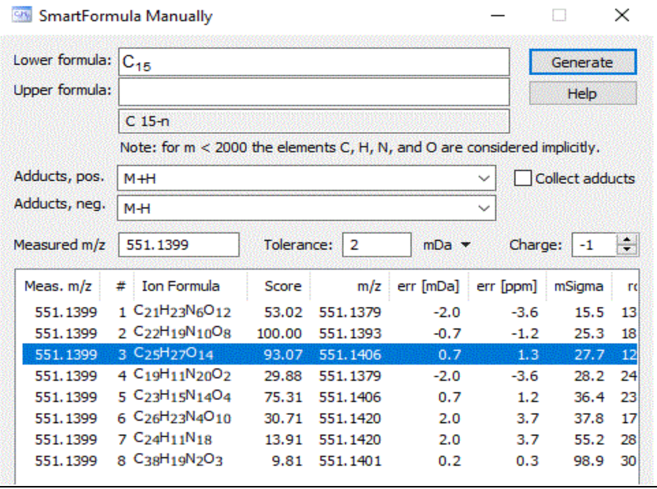

Meas. m/z	#	Ion Formula	Score	m/z	err [mDa]	err [ppm]	mSigma	r
551.1399	1	$C_{21}H_{23}N_6O_{12}$	53.02	551.1379	-2.0	-3.6	15.5	13
551.1399	2	$C_{22}H_{19}N_{10}O_8$	100.00	551.1393	-0.7	-1.2	25.3	18
551.1399	3	$C_{25}H_{27}O_{14}$	93.07	551.1406	0.7	1.3	27.7	12
551.1399	4	$C_{19}H_{11}N_{20}O_2$	29.88	551.1379	-2.0	-3.6	28.2	24
551.1399	5	$C_{23}H_{15}N_{14}O_4$	75.31	551.1406	0.7	1.2	36.4	23
551.1399	6	$C_{26}H_{23}N_4O_{10}$	30.71	551.1420	2.0	3.7	37.8	17
551.1399	7	$C_{24}H_{11}N_{18}$	13.91	551.1420	2.0	3.7	55.2	28
551.1399	8	$C_{38}H_{19}N_2O_3$	9.81	551.1401	0.2	0.3	98.9	30

Fig S4c. Molecular Formula Annotation of m/z 447.0933

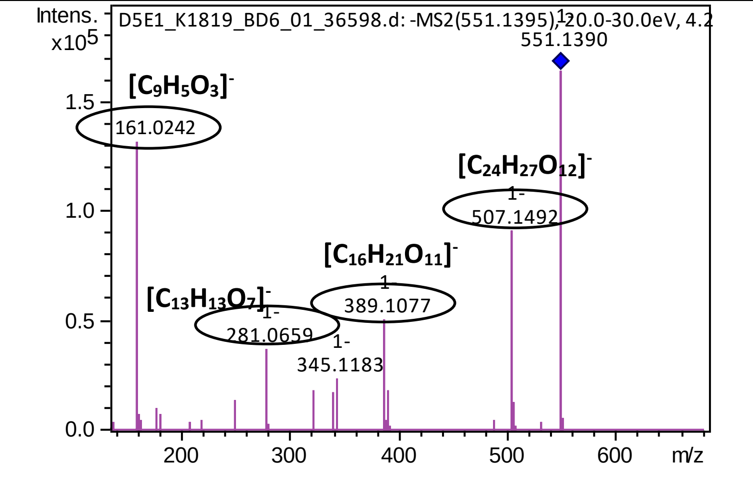

Fig S4d. Background subtracted MS/MS Spectra from 4.2 to 4.3 min

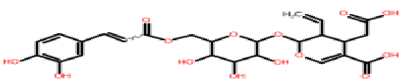

$[C_{25}H_{28}O_{14}]$
Caffeoyl 6-secologanoside

Precursor ion

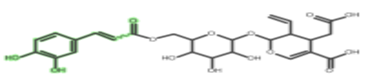

$[C_9H_5O_3]^-$
Mass error: 0.2 mDa

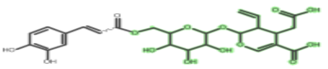

$[C_{16}H_{21}O_{11}]^-$
Mass error: 1.2 mDa

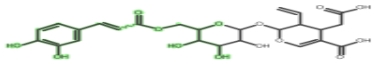

$[C_{13}H_{13}O_7]^-$
Mass error: 0.8 mDa

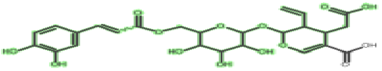

$[C_{24}H_{27}O_{12}]^-$
Mass error: 1.6 mDa

Fig S4e. Structure of precursor and fragment ions of Caffeoyl 6-secologanoside

Figure S4. Identification data for the mass feature m/z 551.1406_4.2 min (caffeoyl 6-secologanoside)

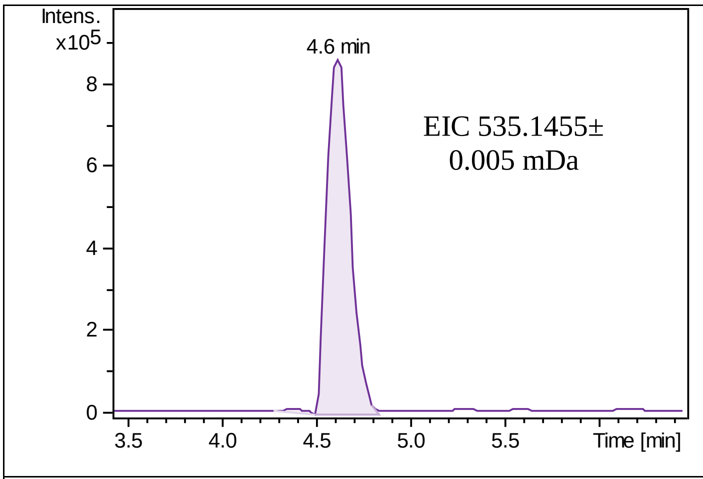

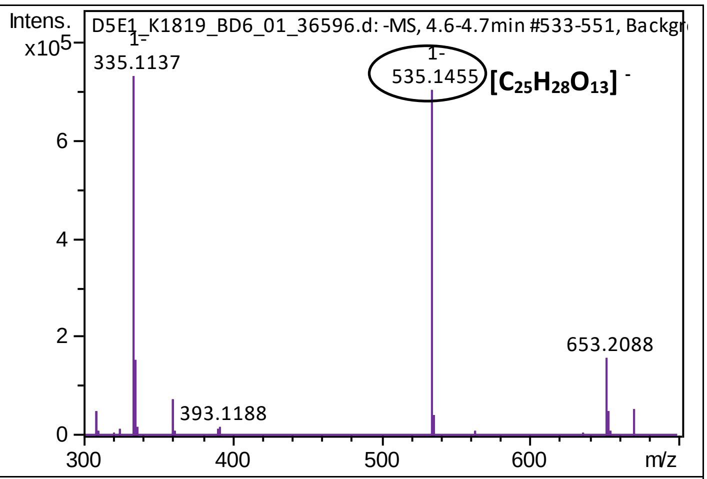

Fig S5a. EIC of m/z 535.1455 in an edible olive fruit

Fig S5b. Background subtracted MS Spectra from 4.5 to 4.7 min

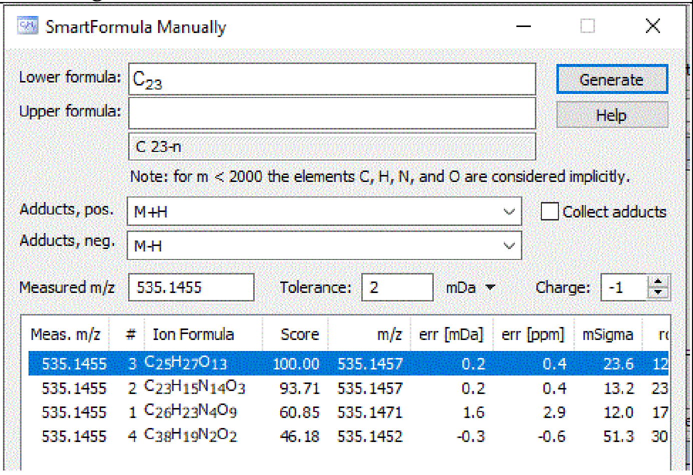

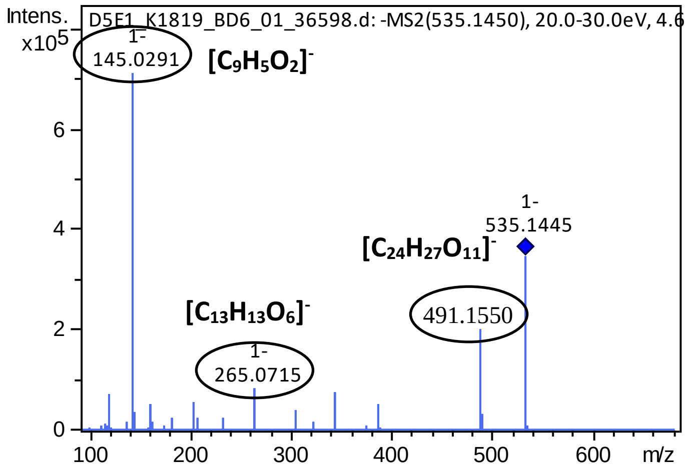

Fig S5c. Molecular Formula Annotation of m/z 535.1455

Fig S5d. Background subtracted MS/MS Spectra from 4.5 to 4.7 min

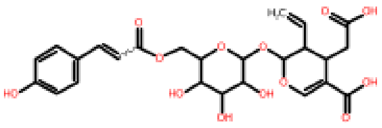

$[C_{25}H_{28}O_{13}]$
Comselogoside
Precursor ion

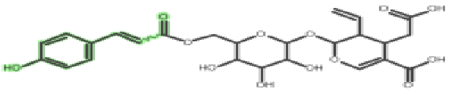

$[C_9H_5O_2]^-$
Mass error: 0.4 mDa

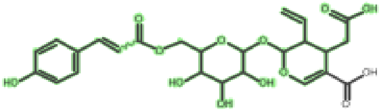

$[C_{24}H_{27}O_{11}]^-$
Mass error: 0.9 mDa

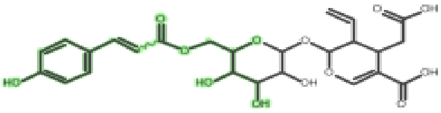

$[C_{13}H_{13}O_6]^-$
Mass error: 0.3 mDa

Fragment ions

Fig S5e. Structures of precursor and fragment ions of Comselogoside

Figure S5. Identification data for the mass feature m/z 535.1455_4.6 min (Comselogoside)

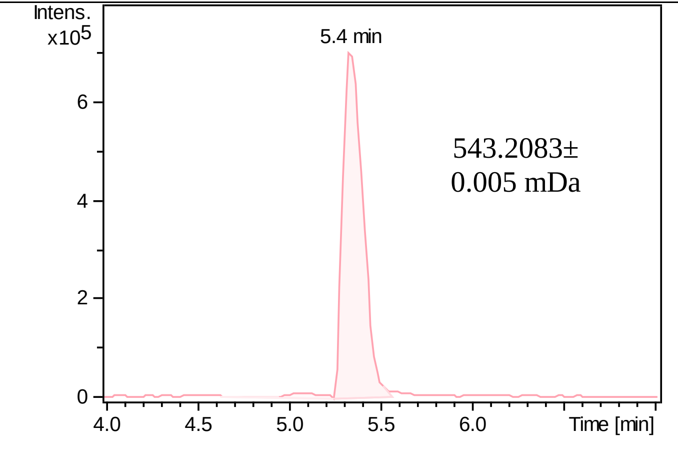

Fig S6a. EIC of m/z 543.2083 in an edible olive fruit

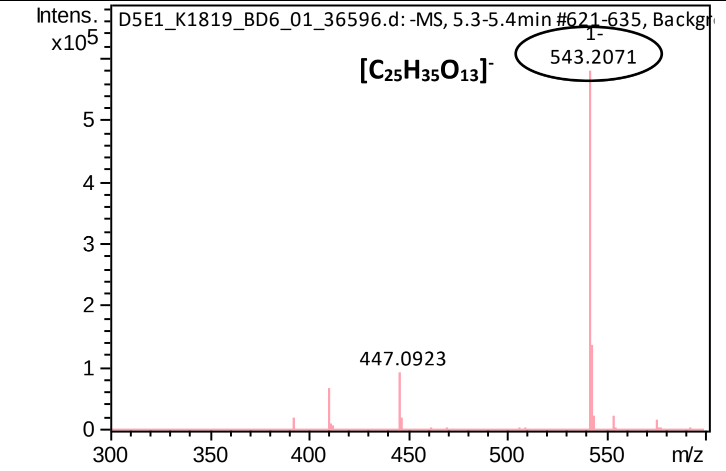

Fig S6b. Background subtracted MS Spectra from 5.3 to 5.5 min

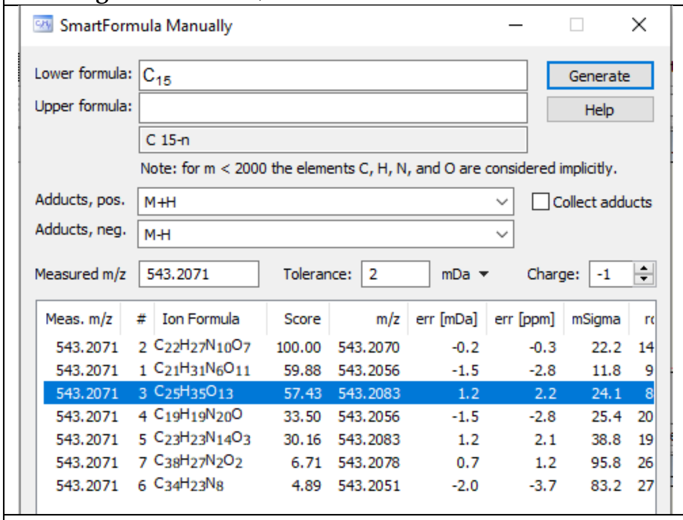

Meas. m/z	#	Ion Formula	Score	m/z	err [mDa]	err [ppm]	mSigma	r
543.2071	2	$C_{22}H_{27}N_{10}O_7$	100.00	543.2070	-0.2	-0.3	22.2	14
543.2071	1	$C_{21}H_{31}N_6O_{11}$	59.88	543.2056	-1.5	-2.8	11.8	9
543.2071	3	$C_{25}H_{35}O_{13}$	57.43	543.2083	1.2	2.2	24.1	8
543.2071	4	$C_{19}H_{19}N_{20}O$	33.50	543.2056	-1.5	-2.8	25.4	20
543.2071	5	$C_{23}H_{23}N_{14}O_3$	30.16	543.2083	1.2	2.1	38.8	19
543.2071	7	$C_{38}H_{27}N_2O_2$	6.71	543.2078	0.7	1.2	95.8	26
543.2071	6	$C_{34}H_{23}N_8$	4.89	543.2051	-2.0	-3.7	83.2	27

Fig S6c. Molecular Formula Annotation of m/z 543.2083

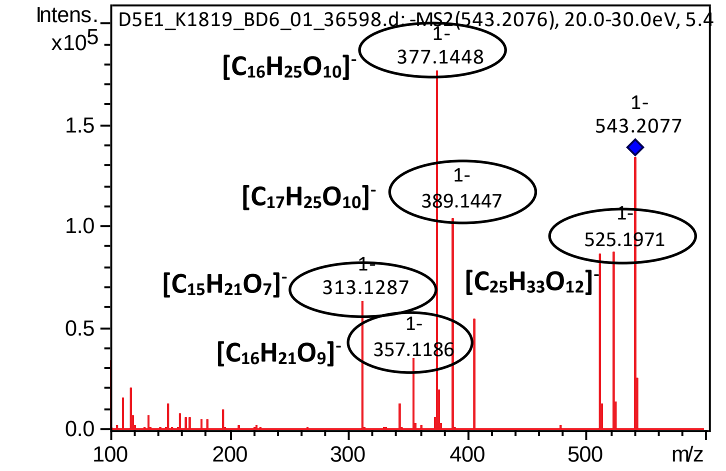

Fig S6d. Background subtracted MS/MS Spectra from 5.3 to 5.5 min

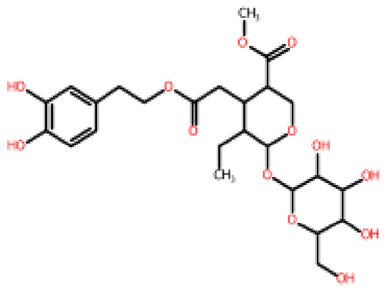

$[C_{29}H_{36}O_{13}]$

dihydrooleuropein

Precursor ion

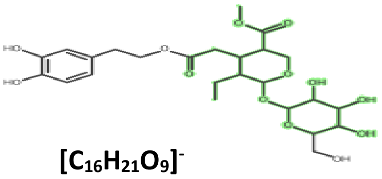

$[C_{16}H_{21}O_9]^-$

Mass error:0.5 mDa

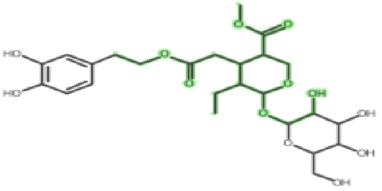

$[C_{15}H_{21}O_7]^-$

Mass error:0.6 mDa

Fragment ions

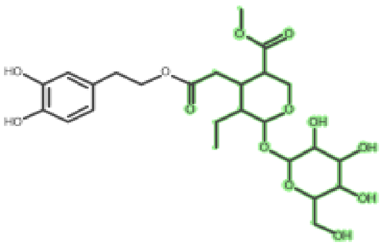

$[C_{17}H_{25}O_{10}]^-$

Mass error:0.6 mDa

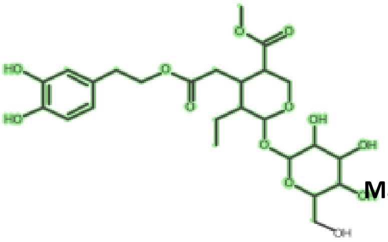

$[C_{25}H_{33}O_{12}]^-$

Mass error: 1.2 mDa

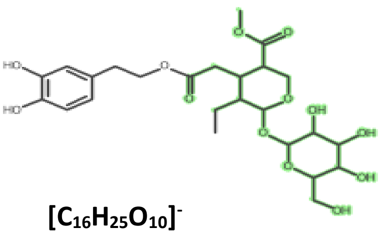

$[C_{16}H_{25}O_{10}]^-$

Mass error:0.6 mDa

Figure S6e. Structures of precursor and fragments ions of dihydrooleuropein

Figure S6. Identification data for the mass feature m/z 543.2083_5.4 min (dihydrooleuropein)

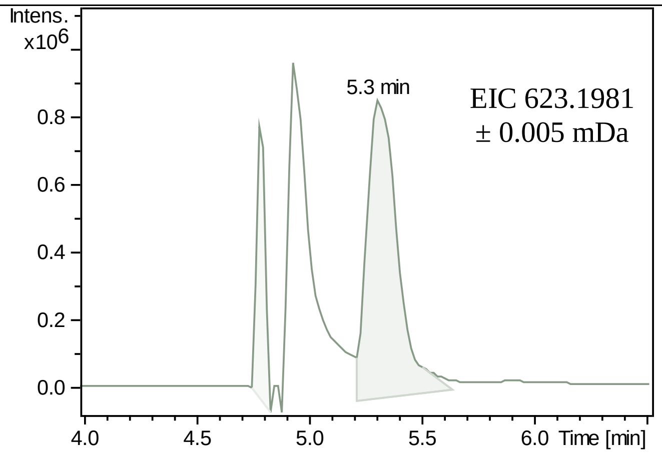

Fig S7a. EIC of m/z 623.1981 in an edible olive fruit

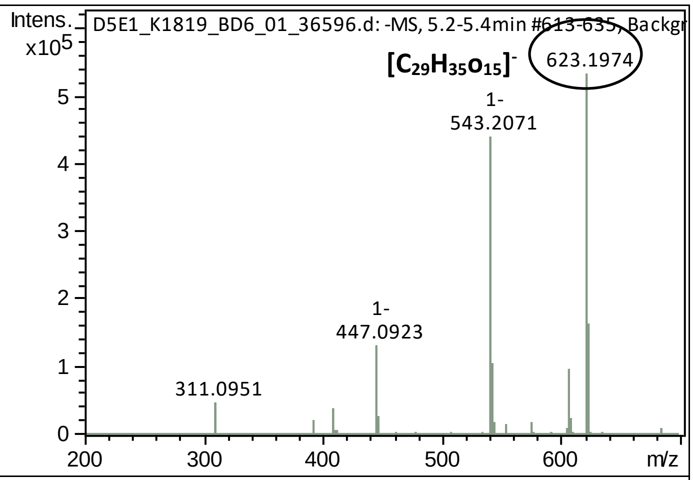

Fig S7b. Background subtracted MS Spectra from 6.3 to 6.5 min

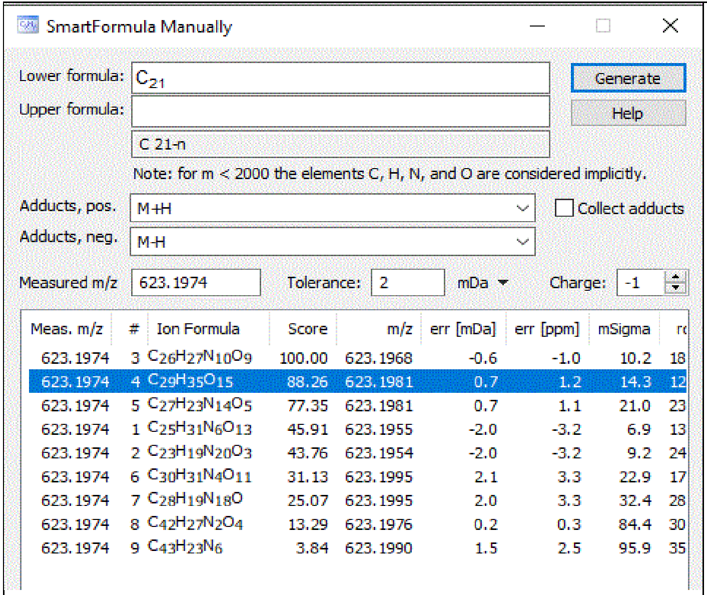

SmartFormula Manually

Lower formula: C_{21}

Upper formula:

C 21-n

Note: for m < 2000 the elements C, H, N, and O are considered implicitly.

Adducts, pos. M+H — Collect adducts

Adducts, neg. M-H

Measured m/z 623.1974 — Tolerance: 2 mDa — Charge: -1

Generate — Help

Meas. m/z	#	Ion Formula	Score	m/z	err [mDa]	err [ppm]	mSigma	rc
623.1974	3	$C_{26}H_{27}N_{10}O_9$	100.00	623.1968	-0.6	-1.0	10.2	18
623.1974	4	$C_{29}H_{35}O_{15}$	88.26	623.1981	0.7	1.2	14.3	12
623.1974	5	$C_{27}H_{23}N_{14}O_5$	77.35	623.1981	0.7	1.1	21.0	23
623.1974	1	$C_{25}H_{31}N_6O_{13}$	45.91	623.1955	-2.0	-3.2	6.9	13
623.1974	2	$C_{23}H_{19}N_{20}O_3$	43.76	623.1954	-2.0	-3.2	9.2	24
623.1974	6	$C_{30}H_{31}N_4O_{11}$	31.13	623.1995	2.1	3.3	22.9	17
623.1974	7	$C_{28}H_{19}N_{18}O$	25.07	623.1995	2.0	3.3	32.4	28
623.1974	8	$C_{42}H_{27}N_2O_4$	13.29	623.1976	0.2	0.3	84.4	30
623.1974	9	$C_{43}H_{23}N_6$	3.84	623.1990	1.5	2.5	95.9	35

Fig S7c. Molecular Formula Annotation of m/z 623.1981

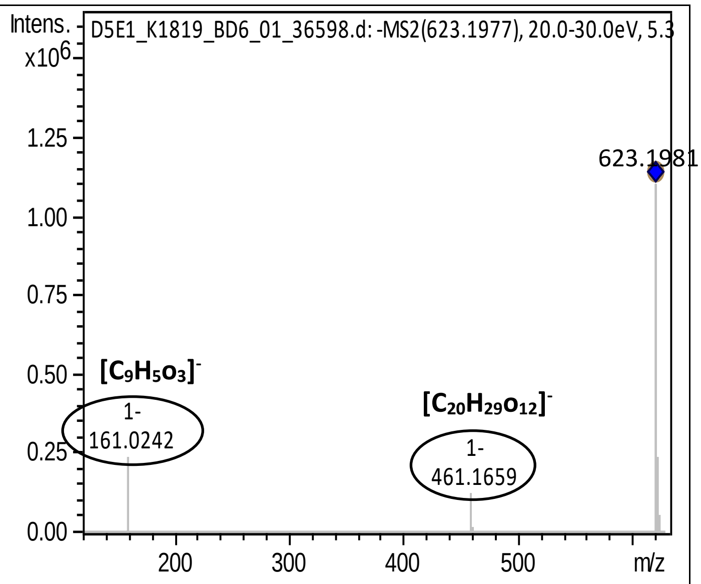

Fig S7d. Background subtracted MS/MS Spectra from 6.3 to 6.5 min

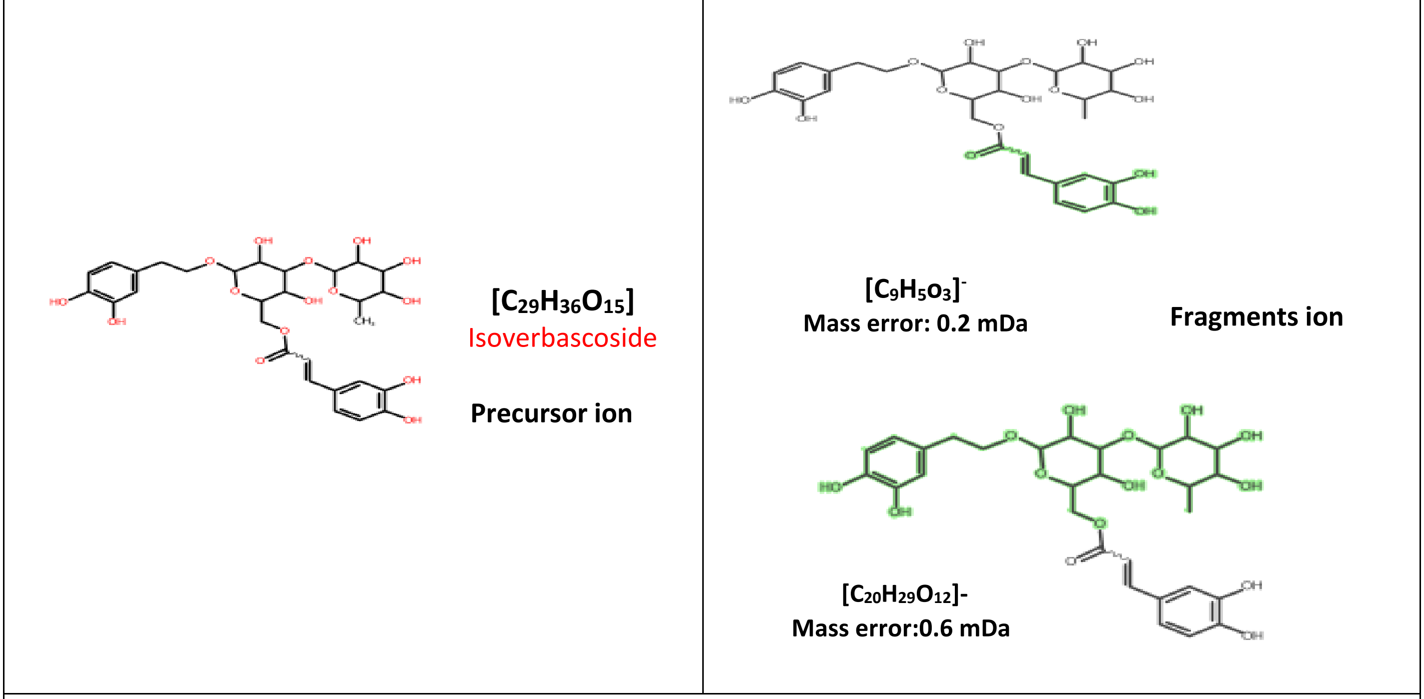

Fig S7e. Structures of precursor and fragment ions of Isoverbascoside

Figure S7. Identification data for the mass feature m/z 623.1981_5.3 min (Isoverbascoside)

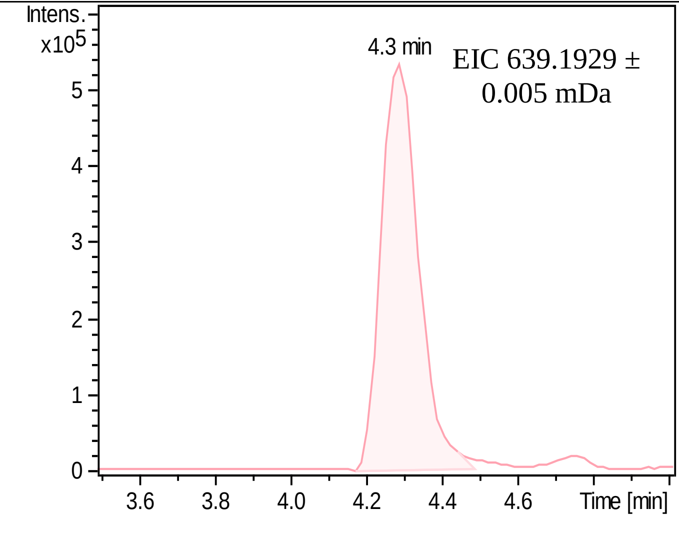

Fig S8a. EIC of m/z 639.1929 in an edible olive fruit

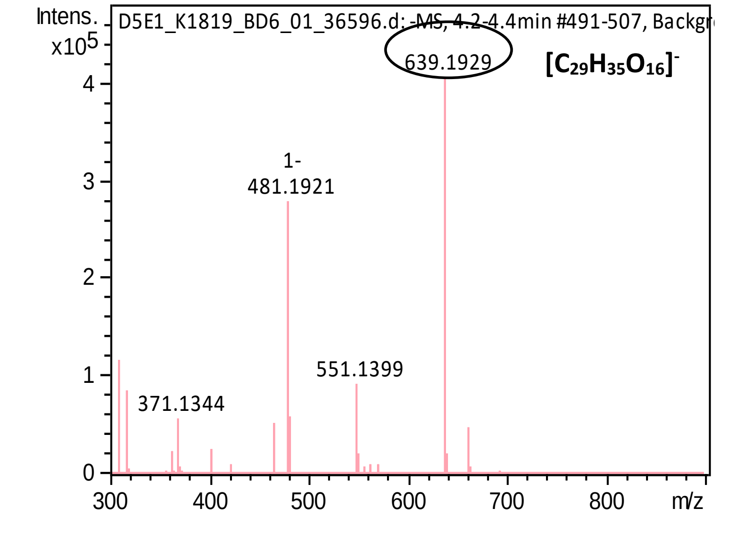

Fig S8b. Background subtracted MS Spectra from 4.0 to 4.2 min

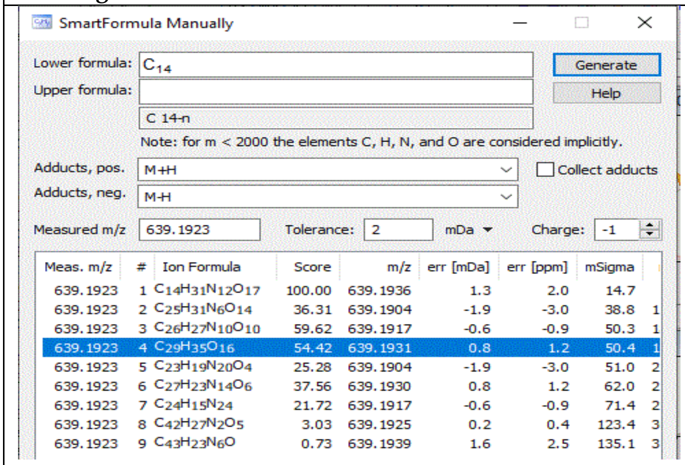
SmartFormula Manually

Lower formula: C14
Upper formula:
C 14-n
Generate
Help
Note: for m < 2000 the elements C, H, N, and O are considered implicitly.
Adducts, pos. M+H
Adducts, neg. M-H
Collect adducts
Measured m/z 639.1923 Tolerance: 2 mDa Charge: -1

Meas. m/z	#	Ion Formula	Score	m/z	err [mDa]	err [ppm]	mSigma	
639.1923	1	$C_{14}H_{31}N_{12}O_{17}$	100.00	639.1936	1.3	2.0	14.7	
639.1923	2	$C_{25}H_{31}N_6O_{14}$	36.31	639.1904	-1.9	-3.0	38.8	1
639.1923	3	$C_{26}H_{27}N_{10}O_{10}$	59.62	639.1917	-0.6	-0.9	50.3	1
639.1923	4	$C_{29}H_{35}O_{16}$	54.42	639.1931	0.8	1.2	50.4	1
639.1923	5	$C_{23}H_{19}N_{20}O_4$	25.28	639.1904	-1.9	-3.0	51.0	2
639.1923	6	$C_{27}H_{23}N_{14}O_6$	37.56	639.1930	0.8	1.2	62.0	2
639.1923	7	$C_{24}H_{15}N_{24}$	21.72	639.1917	-0.6	-0.9	71.4	2
639.1923	8	$C_{42}H_{27}N_2O_5$	3.03	639.1925	0.2	0.4	123.4	3
639.1923	9	$C_{43}H_{23}N_6O$	0.73	639.1939	1.6	2.5	135.1	3

Fig S8c. Molecular Formula Annotation of m/z 639.1929

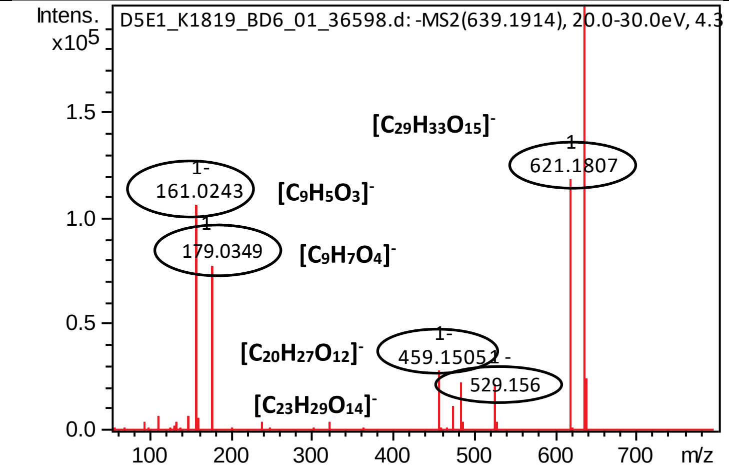

Fig S8d. Background subtracted MS/MS Spectra from 4.0 to 4.2 min

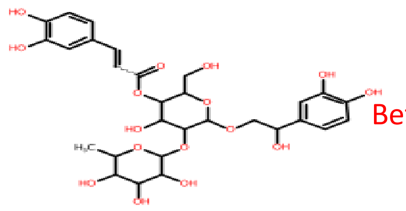

[$C_{29}H_{36}O_{16}$]

Beta hydroxyacteoside

Precursor ion

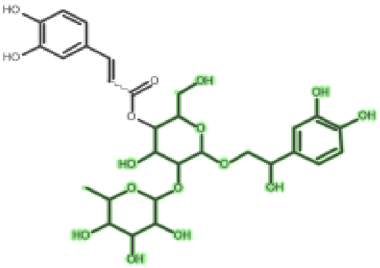

[$C_{23}H_{30}O_{13}$]$^-$
Mass error: 0.3 mDa

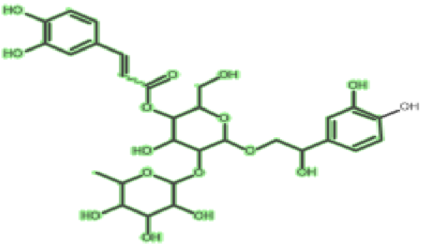

[$C_{29}H_{34}O_{15}$]$^-$
Mass error: 0.1 mDa

Fragment ions

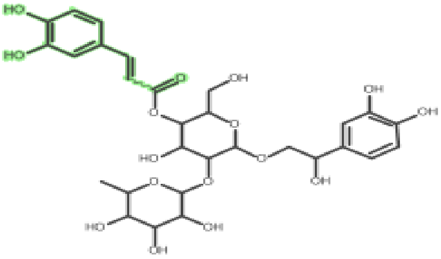

[$C_9H_5O_3$]$^-$
Mass error: 0.1 mDa

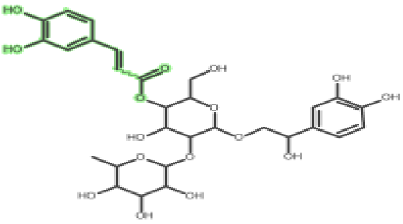

[$C_9H_7O_4$]$^-$
ass error: 0.1 mDa

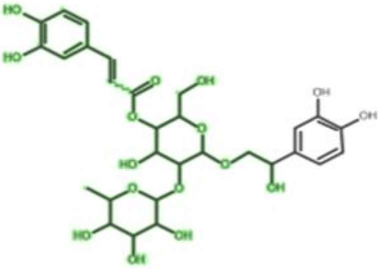

[$C_{20}H_{28}O_{12}$]$^-$
Mass error: 0.3 mDa

Fig S8e. Structures of precursor and fragment ions of Beta- hydroxyacteoside

Figure S8. Identification data for the mass feature m/z 639.1929_4.3 min (beta-hydroxyacteoside)

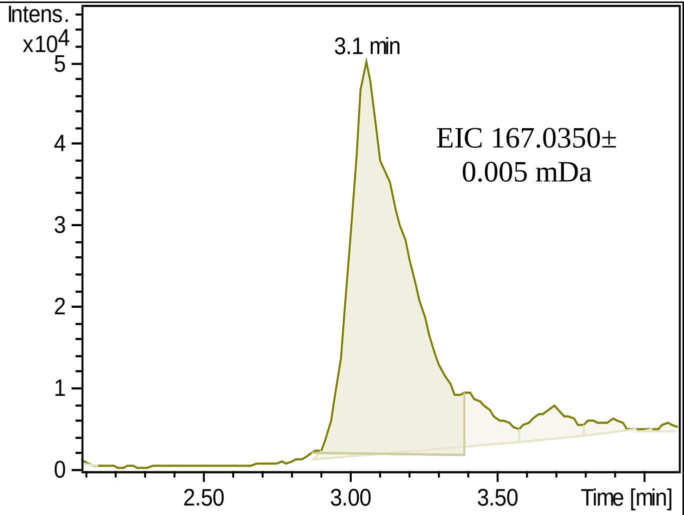

Fig S9a. EIC of m/z 167.0350 in an edible olive fruit

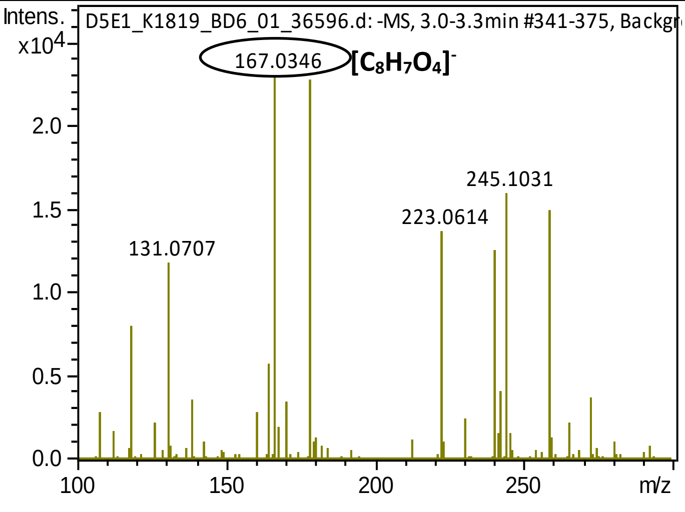

Fig S9b. Background subtracted MS Spectra from 3.0 to 3.3min

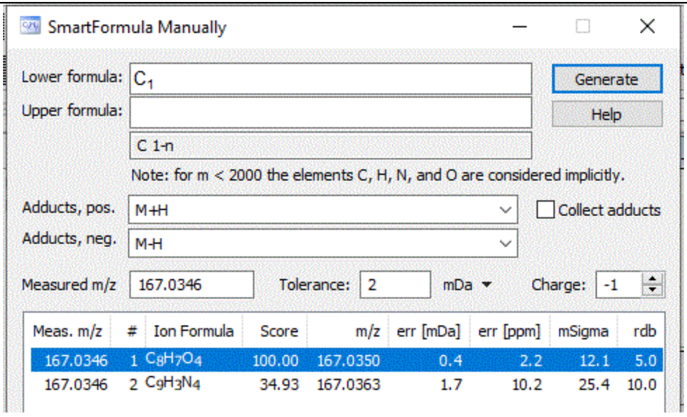

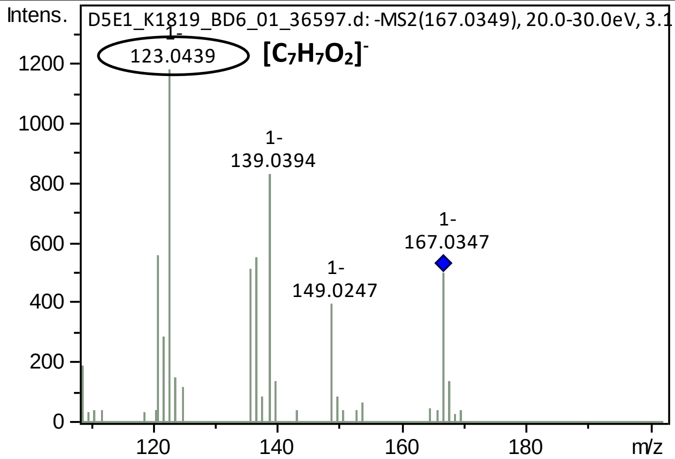

Fig S9c. Molecular Formula Annotation of m/z 167.0350

Fig S9d. Background subtracted MS/MS Spectra from 3.0 to 3.3 min

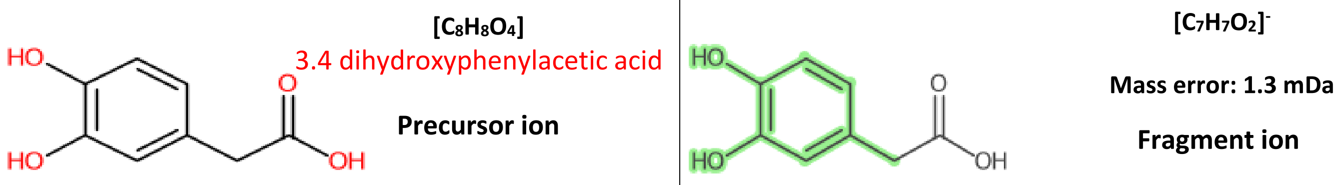

Fig S9e. Structures of precursor and fragment ions of 3.4 dihydroxyphenylacetic acid

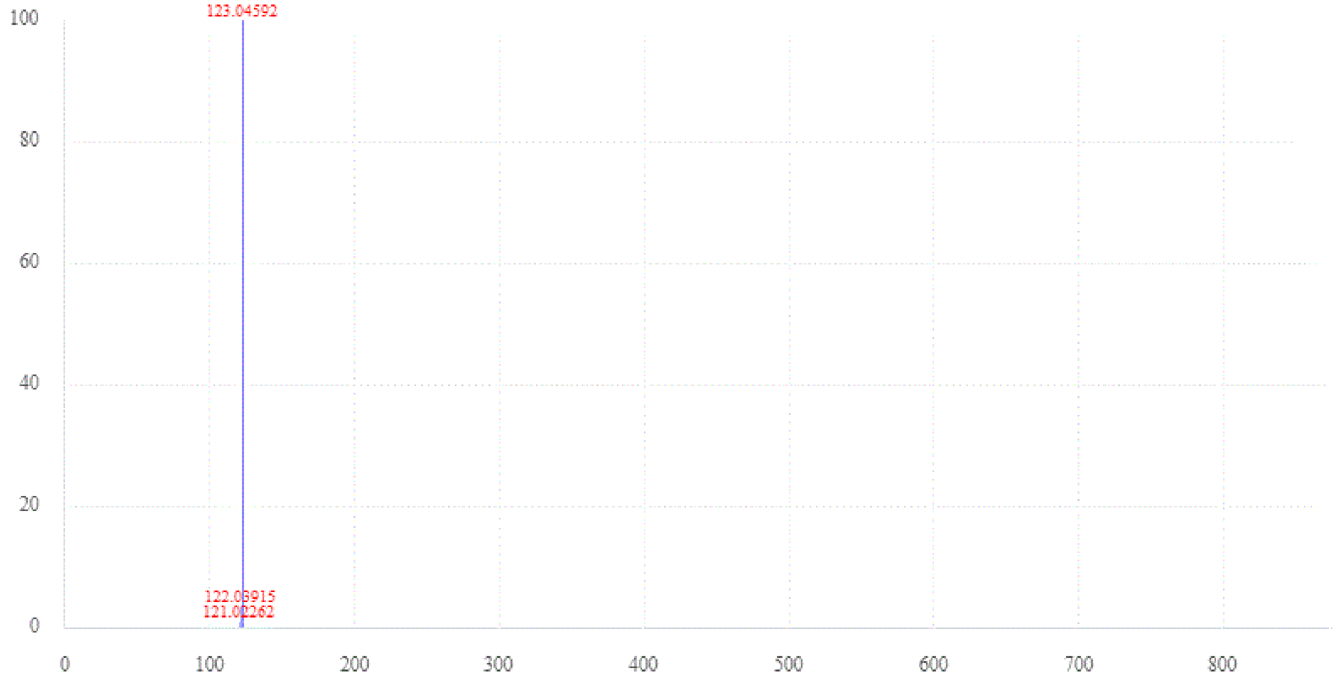

Fig. S9f: Fiehn Lab HILIC Library Record FiehnHILIC002487 (3.4 dihydroxyphenylacetic acid)

Figure S9. Identification data for the mass feature m/z 167.0350_3.1 min (3,4 dihydroxyphenylacetic acid)

Table S7. Maturation stage of Kolovi samples

Sample	Fruit maturity stage (based on color)
**Sample 1**	G. PG. B
**Sample 2**	G
**Sample 3**	G. PG
**Sample 4**	B
**Sample 5**	G. PG
**Sample 6**	G
**Sample 7**	G

G: green. LG. Light Green PG: purple green. B: black

Table S8. LC gradient elution and flow rate program (UPLC-QTOF-MS)

Time (min)	Flow rate (mL/min)	%A	%B
0	0.2	99	1
1.0	0.2	99	1
3.0	0.2	61	39
14.0	0.4	0.1	99.9
16.0	0.48	0.1	99.9
16.1	0.48	99	1
19.0	0.48	99	1
19.1	0.2	99	1
20.0	0.2	99	1

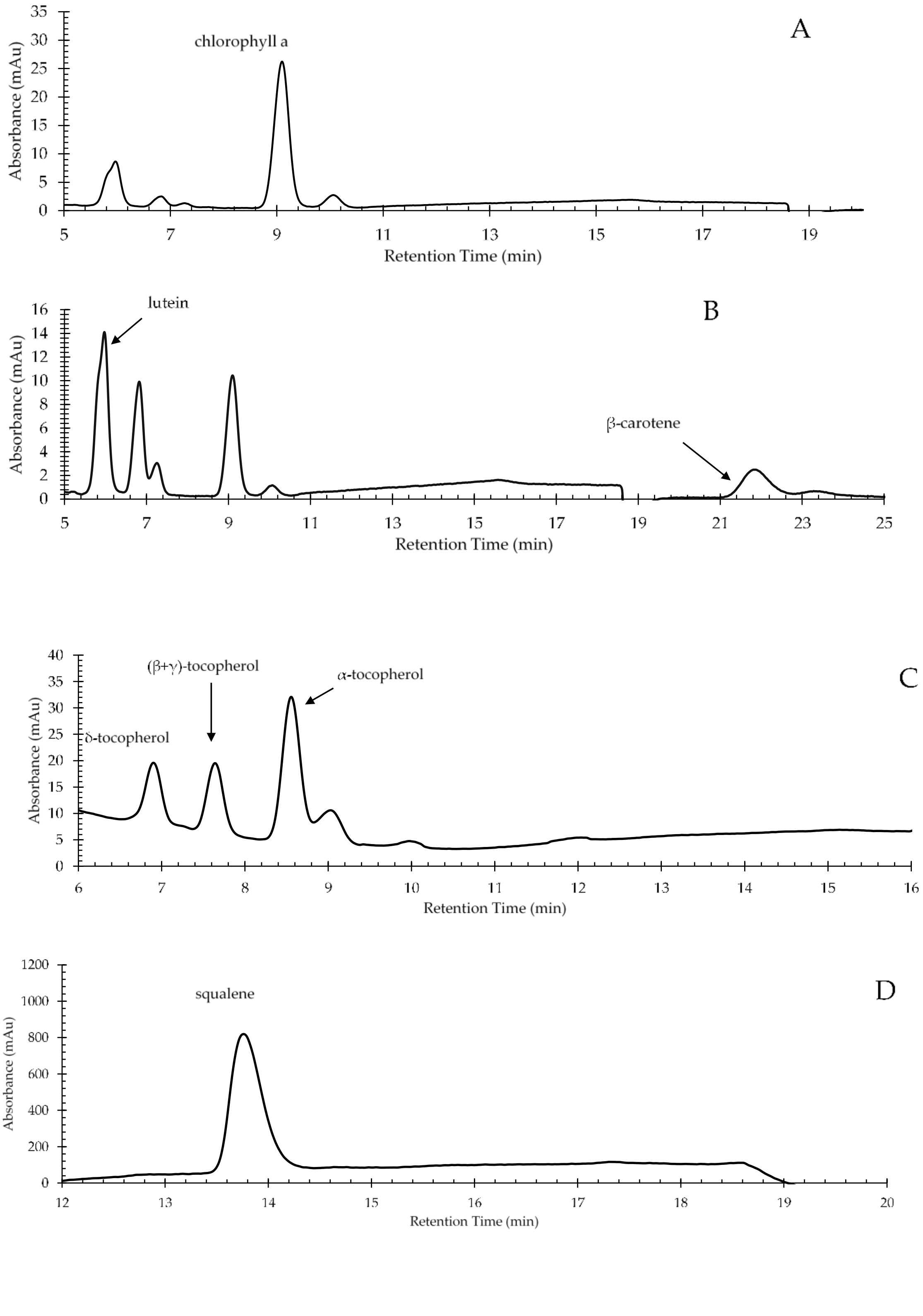

Figure S10: Chromatograms from the analysis of olive drupes. spiked with known amount of the analytes of interest. A: 410 nm (spiked with 25 mg/kg chlorophyll a). B: 450 nm (spiked with 3 mg/kg lutein and 2.95 mg/kg β-carotene). C: 295 nm (spiked with 50 mg/kg α-, γ-, and δ-tocopherols). D: 210 nm (spiked with 500 mg/kg squalene)
